# Effect of the π-conjugation length on the properties and photovoltaic performance of A–π–D–π–A type oligothiophenes with a 4,8-bis(thienyl)benzo[1,2-*b*:4,5-*b*′]dithiophene core

**DOI:** 10.3762/bjoc.12.169

**Published:** 2016-08-10

**Authors:** Ni Yin, Lilei Wang, Yi Lin, Jinduo Yi, Lingpeng Yan, Junyan Dou, Hai-Bo Yang, Xin Zhao, Chang-Qi Ma

**Affiliations:** 1 Printable Electronics Research Center, Suzhou Institute of Nano-Tech and Nano-Bionics (SINANO), Chinese Academy of Sciences, 398 Ruo Shui Road, SEID SIP, Suzhou, Jiangsu, 215123, P. R. China; 2 College of Chemistry, Biology and Material Engineering, Suzhou University of Science and Technology, 1 Ke Rui Road, Suzhou, Jiangsu, 215009, P. R. China; 3 Department of Chemistry, Shanghai Key Laboratory of Green Chemistry and Chemical, East China Normal University, 3663 North Zhongshan Road, Shanghai 200062, P. R. China; 4 Department of Chemistry, Xi’an Jiaotong Liverpool University, 111 Ren Ai Road, Dushu Lake Higher Education Town, Suzhou, Jiangsu, 215123, P. R. China

**Keywords:** A–π–D–π–A-type conjugated molecules, benzodithiophene, π-bridge, chain length effect, organic solar cell

## Abstract

Benzo[1,2-*b*:4,5-*b*′]dithiophene (BDT) is an excellent building block for constructing π-conjugated molecules for the use in organic solar cells. In this paper, four 4,8-bis(5-alkyl-2-thienyl)benzo[1,2-*b*:4,5-*b*′]dithiophene (TBDT)-containing A–π–D–π–A-type small molecules (COOP-*n*HT-TBDT, *n* = 1, 2, 3, 4), having 2-cyano-3-octyloxy-3-oxo-1-propenyl (COOP) as terminal group and regioregular oligo(3-hexylthiophene) (nHT) as the π-conjugated bridge unit were synthesized. The optical and electrochemical properties of these compounds were systematically investigated. All these four compounds displayed broad absorption bands over 350–600 nm. The optical band gap becomes narrower (from 1.94 to 1.82 eV) and the HOMO energy levels increased (from −5.68 to −5.34 eV) with the increase of the length of the π-conjugated bridge. Organic solar cells using the synthesized compounds as the electron donor and PC_61_BM as the electron acceptor were fabricated and tested. Results showed that compounds with longer oligothiophene π-bridges have better power conversion efficiency and higher device stability. The device based on the quaterthiophene-bridged compound **4** gave a highest power conversion efficiency of 5.62% with a *V*_OC_ of 0.93 V, *J*_SC_ of 9.60 mA·cm^−2^, and a FF of 0.63.

## Introduction

Solution-processed organic solar cells (OSCs) are considered to be one of the most promising renewable energy technologies because of the advantages of low cost, lightweight, flexibility, and great potentials in large-scale production [1–2]. In the past few years, OSCs based on polymers have achieved power conversion efficiencies (PCEs) of over 11% [3–4]. Meanwhile, OSCs based on conjugated small molecules attracted also enormous attentions due to their ease of synthesis, defined chemical structure, low batch-to-batch variation, and good reproducibility in photovoltaic performance [5–7]. To date, PCEs of more than 9% for small molecule OSCs (SMOSCs) have been reported [8–12].

Among various electron-donating moieties, benzo[1,2-*b*:4,5-*b*′]dithiophene (BDT) has been widely used as the central building block for constructing high-performance A–π–D–π–A-type organic semiconductors for organic solar cells, where A represents the terminal electron acceptor unit, D represents the core electron donor unit, and π represents the conjugated π-bridge [13–14], and a maximum PCE of 9.95% was reported for a terthiophene-bridged small molecule with a BDT core [8]. Currently, there are three main structure modifications of A–π–D–π–A-type molecules with a BDT core. One is the substitution on the 4,8-positions of the BDT core with aromatic units, including alkyl/alkoxyl/alkylthiol-substituted phenyl groups [15], thienyl group [16–18], and thienothiophene [17,19]. Structure modifications of the BDT core with aromatic units extend the π-conjugation of BDT unit to a two-dimensional structure, which increases intermolecular interactions, and consequently improves the device performance. The other one is to attach different electron acceptor units at the terminal of the molecules, including: dicyanovinyl [20–21], cyanoacetate [20–23], rhodanine [8,14,17,19,23], 1,3-indandione [16], and diketopyrolpyrol (DPP) moieties [24–25]. Changing the electron-withdrawing strength of the terminal electron acceptor unit, on the other hand, will change the intramolecular charge transfer state and tune the light absorption ability, which will consequently change the photovoltaic performance of the materials as well.

The third possible structure modification of BDT derivatives is to tune the conjugation length of the π-bridges. In this respect, oligothiophenes, including monotiophene [16,20], bithiophene [16], terthiophene [8,14–15,17–19,21–23,26], quaterthiophene and quinquethiophene [27], and cyclopentadithiophene [28] have been utilized as the π-bridge in constructing conjugated molecules with a BDT core. Among these 3,3''-dihexyl-2,2':5':2''-terthiophene (3T) is the most widely used π-bridge. It is worth to mention that 3-alkylthiophen can be coupled in different ways at the 2- and 5-positions, yielding oligo(3-alkylthiophene)s with different isomeric structures. In order to minimize the synthesis efforts, structurally symmetric oligothiophene units are mostly used for constructing A–π–D–π–A-type molecules with a BDT core. Interestingly, although various terthiophene-based derivatives with a BDT core have been reported, there is only one paper that reported the synthesis and characterization of BDT derivatives based on oligothiophene π-bridges with more than three thiophene units [27], where symmetric quater- and quinquethiophenes were used as the π-conjugation bridge. Surprisingly, the quaterthiophene-bridged compound showed the worst photovoltaic performance when blending with a fullerene derivative as the photoactive layer. This was ascribed to the influence of the orientation of the alkyl side chains. Although regioregular oligo(3-alkylthiophene)s are better building blocks for studying the effect of the π-conjugation length, only regioregular terthiophene (rr-3T) was reported to be used as the π-bridge unit in BDT derivatives [21–22]. BDT derivatives based on regioregular bi- or quaterthiophene have not been reported, and there is no systematically investigation on the effect of the π-conjugation length yet.

To better understand the effect of the conjugation length on the molecular structure and properties of the conjugated molecules with BDT core, we report here a series of A–π–D–π–A-type conjugated molecules with a regioregular oligo(3-hexylthiophene) chain as the π-bridge unit. The optical and electrochemical properties of these compounds were systematically investigated. Organic solar cells based these conjugated small molecules as the electron donor were fabricated and tested. In addition, long-term stability of these solar cells was also studied, and a general structure–property–performance relationship of these type of molecules is evaluated, which could serve as a useful guideline for further molecular design and synthesis for organic solar cells.

## Results and Discussion

### Synthesis and structure characterization of COOP-nHT-TBDT

**Scheme 1:**
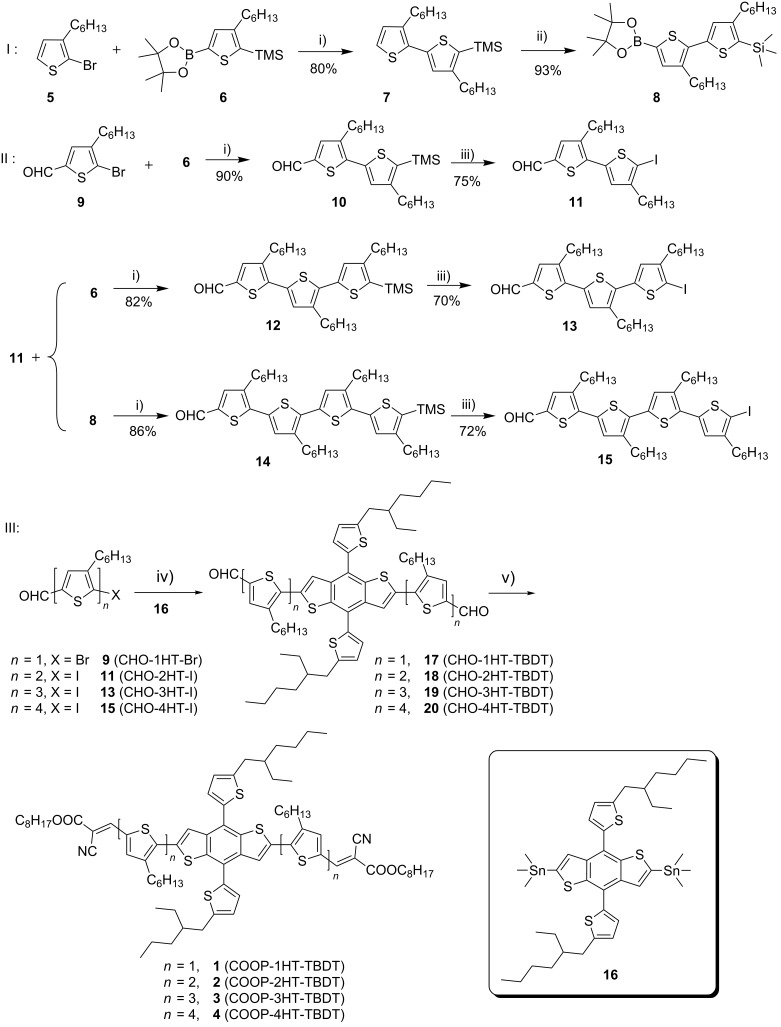
Synthetic route to compounds **1**–**4** with BDT core. Reagents and conditions: i) [Pd_2_(dba)_3_]·CHCl_3_, HP(*t-*Bu)_3_BF_4_, K_2_CO_3_, THF; ii) 1. *n*-BuLi/THF, *2.* 2-isopropyloxy-4,4,5,5-tetramethyl-1,3,2-dioxaborolane; iii) ICl/THF; iv) Pd(PPh_3_)_4_, DMF, 80 °C; v) octyl cyanoacetate, piperidine, CHCl_3_, reflux.

The synthetic routes to compounds **1**–**4** (COOP-*n*HT-TBDT, *n* = 1–4; COOP = 2-cyano-3-octyloxy-3-oxo-1-propenyl, *n*HT = oligo(3-hexylthiophene), TBDT = 4,8-bis(5-alkyl-2-thienyl)benzo[1,2-*b*:4,5-*b*′]dithiophene) is shown in Scheme 1. The bithiophene building block, 3,4'-dihexyl-5'-iodo-2,2'-bithiophene-5-carbaldehyde (**11**) was synthesized by an ipso-substitution of **10**, which was synthesized by a Suzuki coupling of **9** with **6**, with ICl. The regioregular terthiophene and quaterthiophene building blocks were synthesized according to a similar synthetic route starting in high yields. The aldehyde precursors with BDT core CHO-*n*HT-TBDTs **17**–**20** were synthesized through a Pd-catalyzed Stille coupling reaction of **9**, **11**, **13** and **15** with **16**, respectively (reaction iv), and the final compounds, COOP-*n*HT-TBDTs **1**–**4**, were obtained by Knoevenagel condensations of CHO-*n*HT-TBDTs **17**–**20** with octylcyanoacetate (reaction v). Since all these compounds have multiple alkyl chains, they are soluble in common organic solvents, so that the final compounds can be processed well in solution. Complete characterization of both the intermediate and the final compounds was performed by ^1^H NMR, ^13^C NMR and mass spectrometry (see details in Supporting Information File 1).

### Optical properties

**Figure 1:**
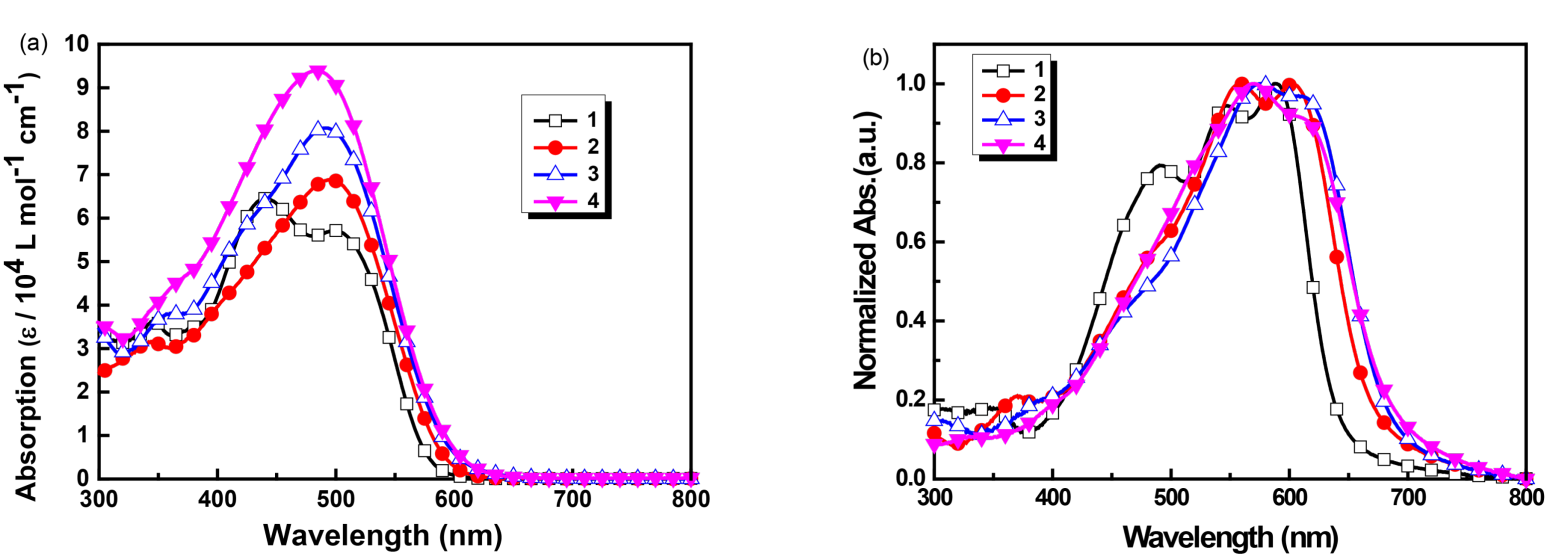
UV−vis absorption spectra of COOP-*n*HT-TBDTs **1**–**4** (a) in chloroform solution (5.0 × 10^−5^ mol·L^-1^) and (b) in solid films.

Figure 1 presents the UV–vis absorption spectra of COOP-*n*HT-TBDTs **1**–**4** in solution and in thin solid films. The spectroscopic data were collected and listed in Table 1. In dilute chloroform solution, all these compounds show intensive absorption bands from 350 to 600 nm with a gradual increase of the molar extinction coefficient. Interestingly, the three molecules **2**, **3**, and **4** display one broad absorption band peaking at 494, 491, and 485 nm, respectively, while two absorption bands peaking at 440 and 498 nm were found for **1**. Elongation of the π-conjugated bridge leads to a slight hypochromic shift of the absorption band, presumably ascribed to a disorder of the complex structures of the π-conjugation chain, or due to the steric hindrance effects of the alkyl side chains for the bigger molecules [29–30]. The absorption onset wavelength increases slightly with the increase of the π-bridge chain length, suggesting an extended π-conjugation system for the compounds with longer oligothiophene chains.

**Table 1:** Optical and electrochemical properties of **1**–**4** in comparison to other compounds in the literature.

compound	*λ*_max_^sol^ [nm]^a^	ε_max_^sol^ [mol^−1^·L·cm^−1^]^b^	*λ*_max_^film^ [nm]	*E*_g_^opt^ [eV]^c^	*E*^0^_ox_ [V]^d^	*E*^0^_red_ [V]^d,e^	*E*_HOMO_ [eV]^f^	*E*_LUMO_ [eV]^f^	*E*_g_^cv^ [eV]^g^	ref.

**1**	440 (498)^h^	64,500	580 (547)^h^	1.94	0.66	−1.53	−5.68 (−5.38)^i^	−3.65 (−3.35)^i^	2.03	[20]
**2**	494	68,800	558 (602)^h^	1.86	0.50	−1.62	−5.52 (−5.22)^i^	−3.56 (−3.26)^i^	1.96	this work
**3**	491	80,700	576 (625)^h^	1.82	0.42	−1.63	−5.43 (−5.13)^i^	−3.55 (−3.25)^i^	1.88	this work
**4**	485	93,900	570 (614)^h^	1.82	0.32	−1.64	−5.35 (−5.05)^i^	−3.53 (−3.23)^i^	1.82	this work
DCAO3TBDT	494	72,000	560	1.84	—	—	−5.04^i^	−3.24^i^	1.80	[23]
TBDTCNR	488	—	578	1.75	—	—	−5.40^i^	−3.63^i^	1.77	[21]

^a^In CHCl_3_ (5.0 × 10^−5^ mol·L^−1^); ^b^extinction coefficient was obtained by linearly fitting the absorbance as a function of the concentration; ^c^optical band gap, calculated from the absorption onset wavelength (λ_onset_) in solid films according to the equation *E*_g_^opt^ (eV) = 1240/λ_onset_ (nm); ^d^measured in CHCl_3_ solution (1.0 × 10^−3^ mol·L^−1^); ^e^irreversible wave: *E*^0^_red_ was estimated as the potential where *i*_pc_ = 0.855 × *i*_pc_^max^; ^f^calculated from the cyclic voltammograms, *E*_HOMO_ = −[*E*_ox_^onset^ + 5.1] (eV), *E*_LUMO_ = −[*E*_red_^onset^ + 5.1] (eV); ^g^electrochemical band gap *E*_g_^cv^= *E*_HOMO_ − *E*_LUMO_ = −[*E*_ox_^onset^ − *E*_red_^onset^] (eV); ^h^shoulder peak; ^i^calculated from the cyclic voltammograms, *E*_HOMO_ = −[*E*_ox_^onset^ + 4.8] (eV), *E*_LUMO_ = −[*E*_red_^onset^ + 4.8] (eV).

In the solid state, absorption spectra of these compounds (see Figure 1b) are remarkably broadened and red-shifted relative to those in solutions, which is attributable to strong π–π stacking interaction between the molecular backbones in the solid films [31–32]. It is noticeable that compound **1** in the film shows two shoulder peaks in the long wavelength range, while compounds **2**, **3**, and **4** have only one shoulder peak. The optical band gaps calculated from the onsets of absorption edge of the four molecules are 1.94, 1.86, 1.82 and 1.82 eV, respectively, in agreement with the BDT derivatives reported in the literatures [21,23].

### Electrochemical properties

**Figure 2:**
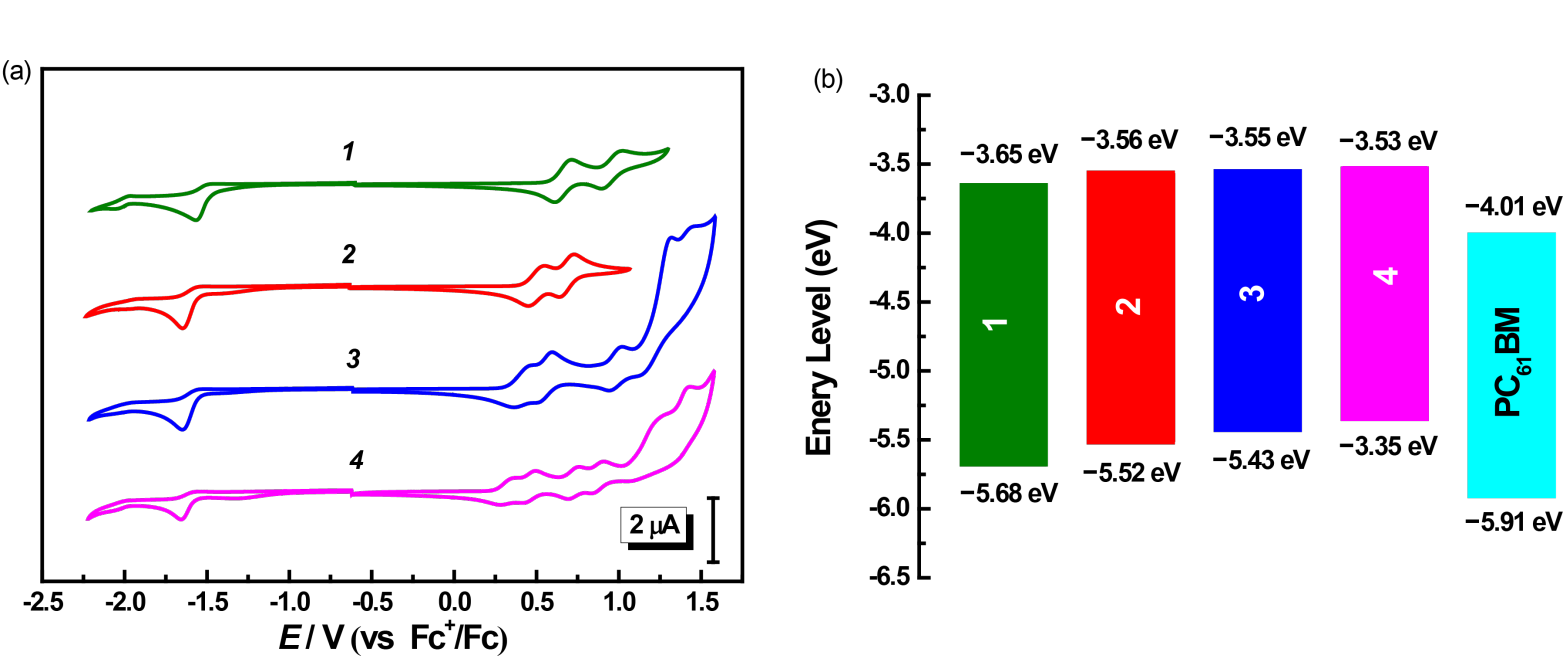
(a) Cyclic voltammograms of **1**–**4** measured in CH_2_Cl_2_ solution (1.0 × 10^−3^ mol·L^−1^) with 0.1 mol·L^−1^ Bu_4_NPF_6_ at a scan rate of 100 mV·s^−1^; (b) HOMO and LUMO energy levels of these compounds.

Cyclic voltammetry (CV) was applied to investigate the energy levels of **1**–**4**. The cyclic voltammograms of these four compounds are presented in Figure 2 and the electrochemical data are listed in Table 1. As can be seen from Figure 2, compounds **1** and **2** exhibited two reversible oxidation processes in the positive range, while **3** and **4** showed multiple oxidation processes, suggesting more oxidation processes of the π-conjugation bridge units for the larger molecules. The first oxidation potentials (*E*^0^_ox_ vs Fc^+^/Fc) for COOP-*n*HT-TBDTs were measured to be 0.66, 0.50, 0.42, and 0.32 V, for *n* = 1, 2, 3, and 4, respectively, indicating a decreasing trend with the increase of the π-bridge chain length. Meanwhile, one irreversible reduction process was found for all these four compounds in the negative potential range. Except for **1**, which showed a reduction potential (*E*^0^_red_) of −1.53 eV, the other three compounds showed almost identical *E*^0^_red_ of −1.60 V, indicating that the reduction process is mainly due to the reduction of the terminal COOP group. The onset oxidation potentials (*E*_ox_^onset^) and onset reduction potentials (*E*_red_^onset^) determined from the CV results are also listed in Table 1. The frontier molecular orbital energy levels (HOMO/LUMO) and also the energy band gaps of these compounds were calculated according to the method reported in our previous paper [20], where the ferrocene/ferrocenium couple (Fc^+^/Fc) was used as the standard, and the vacuum energy level of Fc^+^/Fc was taken as −5.1 eV [33]. As can be seen from this table, the HOMO energy levels of COOP-*n*HT-TBDTs increased slightly from −5.68 to −5.34 V with increasing π-conjugation bridge length. Except for **1**, which has a LUMO level of −3.65 eV, the other three compounds possess similar LUMO level at −3.55 eV, attributed to the same electron-withdrawing terminal units. The LUMO energy levels of COOP-*n*HT-TBDTs are more than 0.3 eV higher than that of PC_61_BM [34], which provides a sufficient driving force for electron transfer from COOP-*n*HT-TBDTs to PC_61_BM (Figure 2b). On the other hand, the low-lying HOMO energy level of COOP-*n*HT-TBDTs would be beneficial for achieving a high open circuit voltage (*V*_OC_), since *V*_OC_ of organic solar cells is directly related to the difference of the HOMO energy of the donor material and the LUMO energy of the acceptor.

### Photovoltaic performance

**Figure 3:**
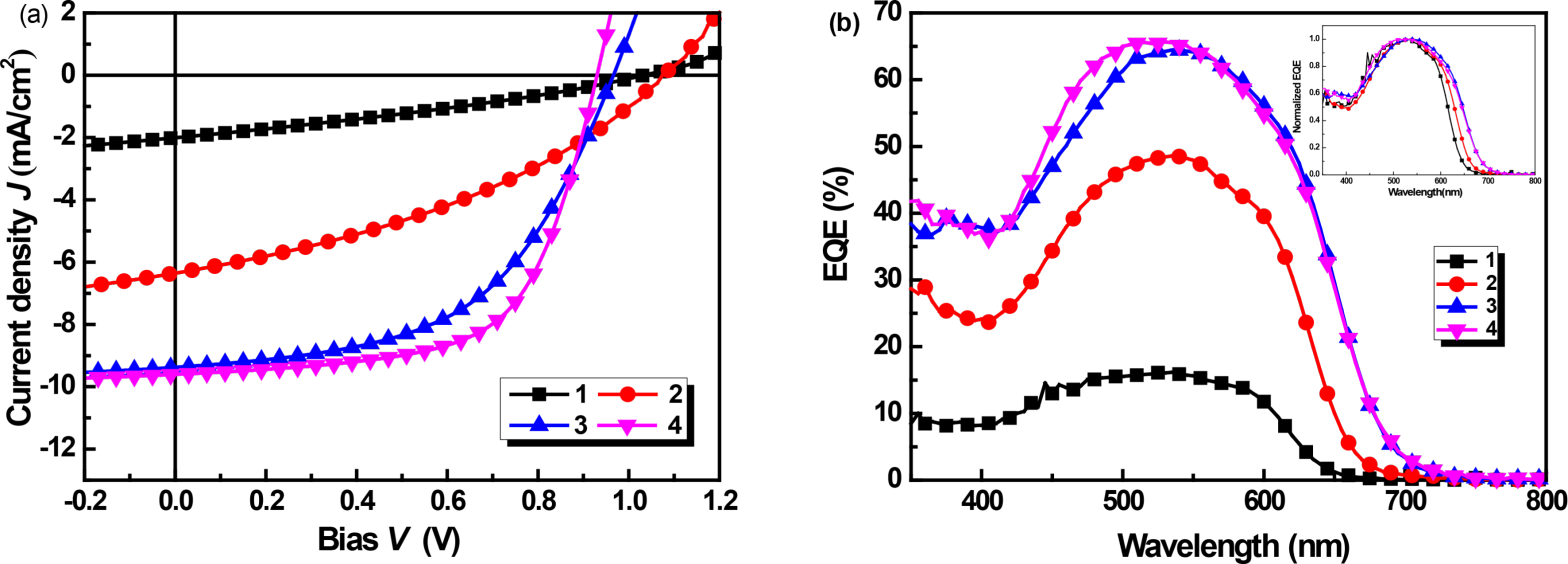
(a) *J*–*V* curves of the best COOP-*n*HT-TBDT:PC_61_BM solar cells; (b) EQE spectra of the corresponding cells, Inset: normalized EQE spectra of four best devices, showing the difference in spectrum response wavelength range.

Bulk heterojunction (BHJ) solar cells with a device structure of ITO/PEDOT:PSS (30 nm)/photoactive layer/LiF (1.5 nm)/Al (100 nm) were fabricated and tested, where the blended solid film of the synthesized small molecules as donor and PC_61_BM as acceptor was used as the photoactive layer. The photovoltaic performances of **1** has been reported in our previous paper [20], and the PV performance data of the best cell are listed in Table 2 for comparison. The photovoltaic performance of cells based on compounds **2**, **3**, and **4** were carefully optimized by varying the D/A blend ratio. Figure S4 and S5 (Supporting Information File 1) depict the current density–voltage (*J*–*V*) curves and the external quantum efficiency (EQE) spectra of cells based on **2**, **3**, and **4**, and the photovoltaic performance data are listed in Table 2. As can be seen from Table 2, the optimal D/A blend ratio for **2**:PC_61_BM based cells was found to be 1:0.6, which showed a maximum PCE of 2.52% (and an average PCE of 2.35%) with a high *V*_OC_ of 1.07 V, *J*_SC_ of 6.36 mA·cm^−2^, and FF of 37% for the best cell. The optimal D/A blend ratio was found to be 1:0.4 for cells based on **3** and **4** (Table 2, entry 6 and 10), which is different to that of cells based on **1** and **2**. High PCEs of 4.73% and 5.62% were achieved for cells with **3** and **4**, respectively, which are much higher than that of the devices based on smaller molecules. Obviously, with the extension of conjugation π-bridges, the PV performance of the COOP-*n*HT-TBDTs enhanced gradually. Since *V*_OC_ decreases slightly with the increase of the conjugation length of the π-bridge, such a device performance enhancement was mainly ascribed to the increase of *J*_SC_ and FF (Table 2). EQE spectra comparison clearly confirmed the higher photon-to-electron conversion efficiency for the bigger molecules (Figure 3b). In addition, the photo responses of devices based on **3** and **4** cover a wavelength range from 380 to 700 nm, which is wider than that of devices based on **1** and **2** (Figure 3b, insert), agreeing with the absorption spectra of the corresponding thin solid films (Figure 1b). Solar cells based on a **4**:PC_71_BM photoactive layer were also fabricated and tested. However, the PC_71_BM-based devices showed a slightly decreased performance compared to the PC_61_BM-based devices (Figure S5, Supporting Information File 1). Using additives or post-thermal annealing did not improve device performance. We speculate that impurities in PC_71_BM or the non-ideal interface between PEDOT:PSS and the photoactive layer could be the reason for the lower device performance. However, further experiments are still need to fully understand the detailed reasons. Nevertheless, the PCE of 5.62% for the **4**:PC_61_BM cells is among the best performance for cells based on COOP-capped BDT derivatives [21–23,35–37].

**Table 2:** Photovoltaic properties of COOP-*n*HT-TBDT:PC_61_BM-based devices.

entry	donor	D/A ratio [w/w]	*V*_OC_ [V]	*J*_SC_ [mA·cm^−2^]^a^	FF [%]	PCE [%]	average PCE [%] (± std. dev.)^b^

1	**1**^c^	1:0.6	1.04	2.28	29	0.69	0.61 (± 0.072)

2	**2**	1:0.4	1.04	5.30	36	2.01	1.89 (± 0.24)
3		1:0.6	1.07	6.36	37	2.52	2.35 (± 0.10)
4		1:0.8	1.06	5.20	36	1.98	1.78 (± 0.17)

5	**3**	1:0.2	0.97	5.99	47	2.73	2.64 (± 0.08)
6		1:0.4	0.97	9.38	52	4.73	4.58 (± 0.16)
7		1:0.6	0.97	8.94	47	4.07	3.79 (± 0.18)
8		1:0.8	0.95	6.35	37	2.23	1.97 (± 0.21)

9	**4**	1:0.2	0.93	6.07	59	3.33	3.14 (± 0.15)
10		1:0.4	0.93	9.60	63	5.62	5.27 (± 0.21)
11		1:0.6	0.91	7.73	44	3.07	2.75 (± 0.27)
12		1:0.8	0.91	5.72	40	2.08	1.93 (± 0.21)

^a^determined by convoluting the spectral response with the AM 1.5G spectrum (100 mW·cm^−2^); ^b^standard deviation was calculated over eight individual devices; ^c^data from [20].

### Surface morphology of the blended films

**Figure 4:**
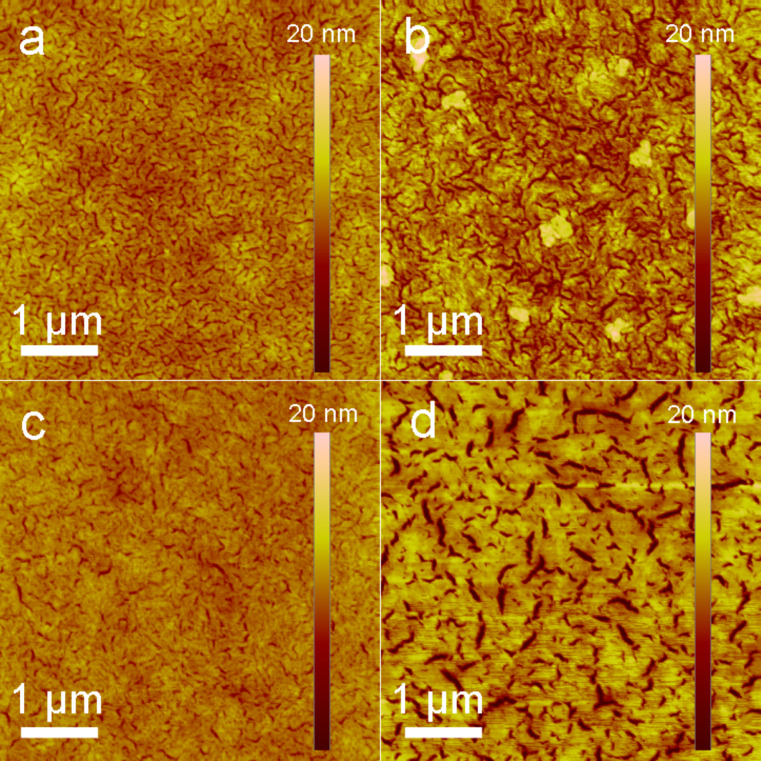
AFM height images of COOP-*n*HT-TBDT:PC61BM blended films: (a) **1**, 1:0.6 (w/w); (b) **2**, 1:0.6 (w/w); (c) **3**, 1:0.4 (w/w); (d) **4**, 1:0.4 (w/w).

The surface morphology of the COOP-*n*HT-TBDT:PC_61_BM films was scrutinized with atomic force microscopy (AFM). Figure 4 depicts the topological images of the blended films prepared under the optimized conditions. The surface roughness for the COOP-*n*HT-TBDT:PC_61_BM blended films was measured to be 1.23, 2.14, 0.96 and 2.84 nm for films based on **1**, **2**, **3**, and **4**, respectively, demonstrating a reasonable surface smoothness for these films. Obviously, crystalline domains can be seen in these films, among which, the **2**:PC_61_BM and **4**:PC_61_BM blended films showed larger crystalline domains compared to the films based on **3** and **4**. Such a nanomorphology difference could be ascribed to the chemical structure difference of the π-conjugation bridges, demonstrating a possible odd–even effect. Nevertheless, the large crystalline domains of the **4**-based film lead to a better charge carrier mobility (vide infra), which is beneficial for device performance.

### Charge-carrier mobility of the blended films

**Figure 5:**
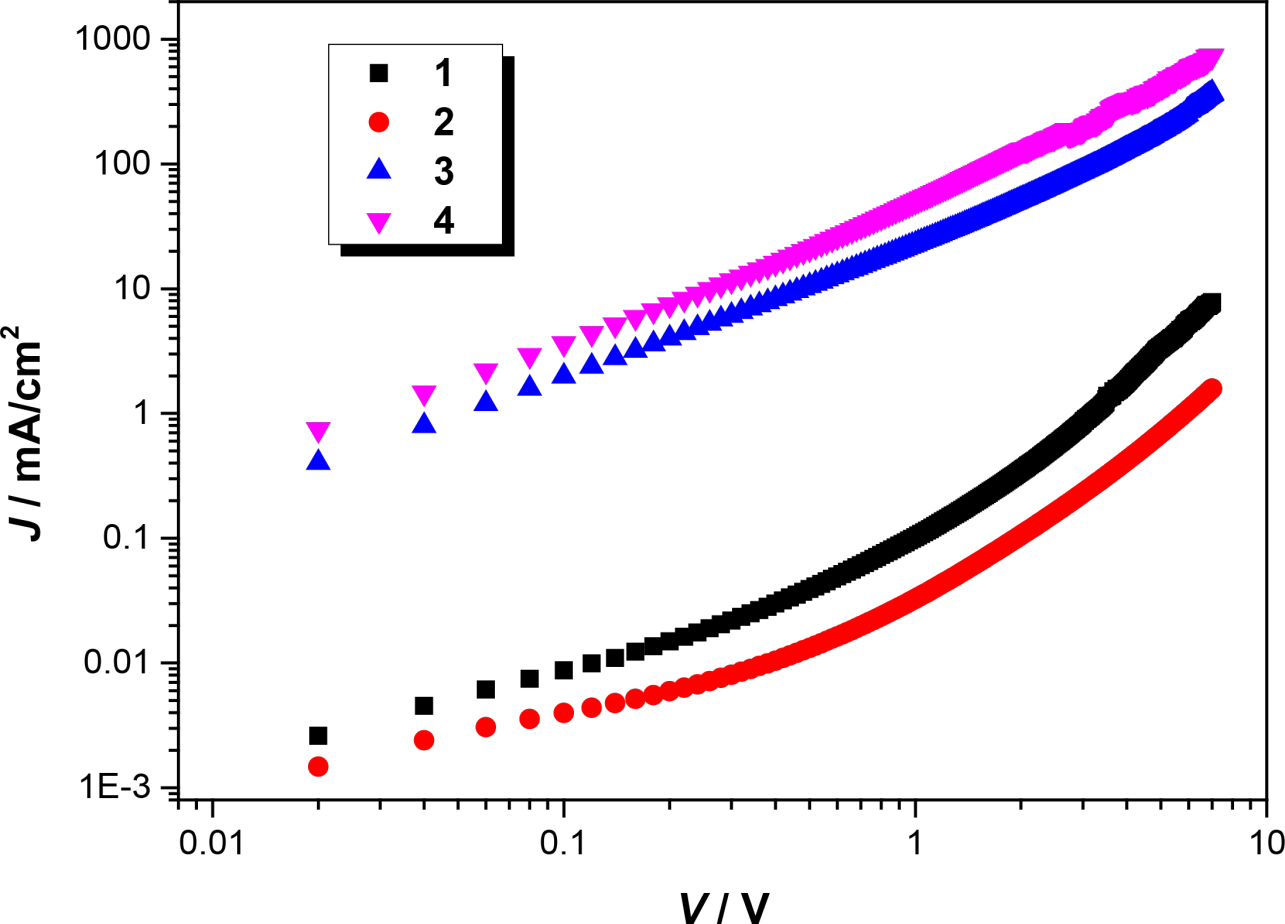
*J*–*V* curves of COOP-*n*HT-TBDT:PC_61_BM-based hole-only devices.

To further understand the influence of the chemical structure on the device performance, the hole mobility of these compounds in blended films was measured using the space-charge-limited current method (SCLC). The device structure studied here was ITO/PEDOT:PSS/COOP-*n*HT-TBDT:PC_61_BM/MoO_3_/Al, and the thin film deposition method is similar to that for solar cell fabrication. The analysis method was described in detail in our previous paper [34]. The hole transport mobilities of COOP-*n*HT-TBDT were measured to be 2.01 × 10^−6^, 1.81 × 10^−6^, 4.60 × 10^−4^ and 8.22 × 10^−4^ cm^2^·V^−1^·s^−1^ for **1**, **2**, **3**, and **4**, respectively. Obviously, the largest molecule **4** displays the highest hole mobility, which could be owing to the formation of large crystalline domains in **4**:PC_61_BM blended film, as shown in Figure 5. The high hole mobility for the larger molecules could be one of the reasons for the higher power conversion efficiency for devices based on COOP-*n*HT-TBDT:PC_61_BM (Table 2).

### Long-term stability

**Figure 6:**
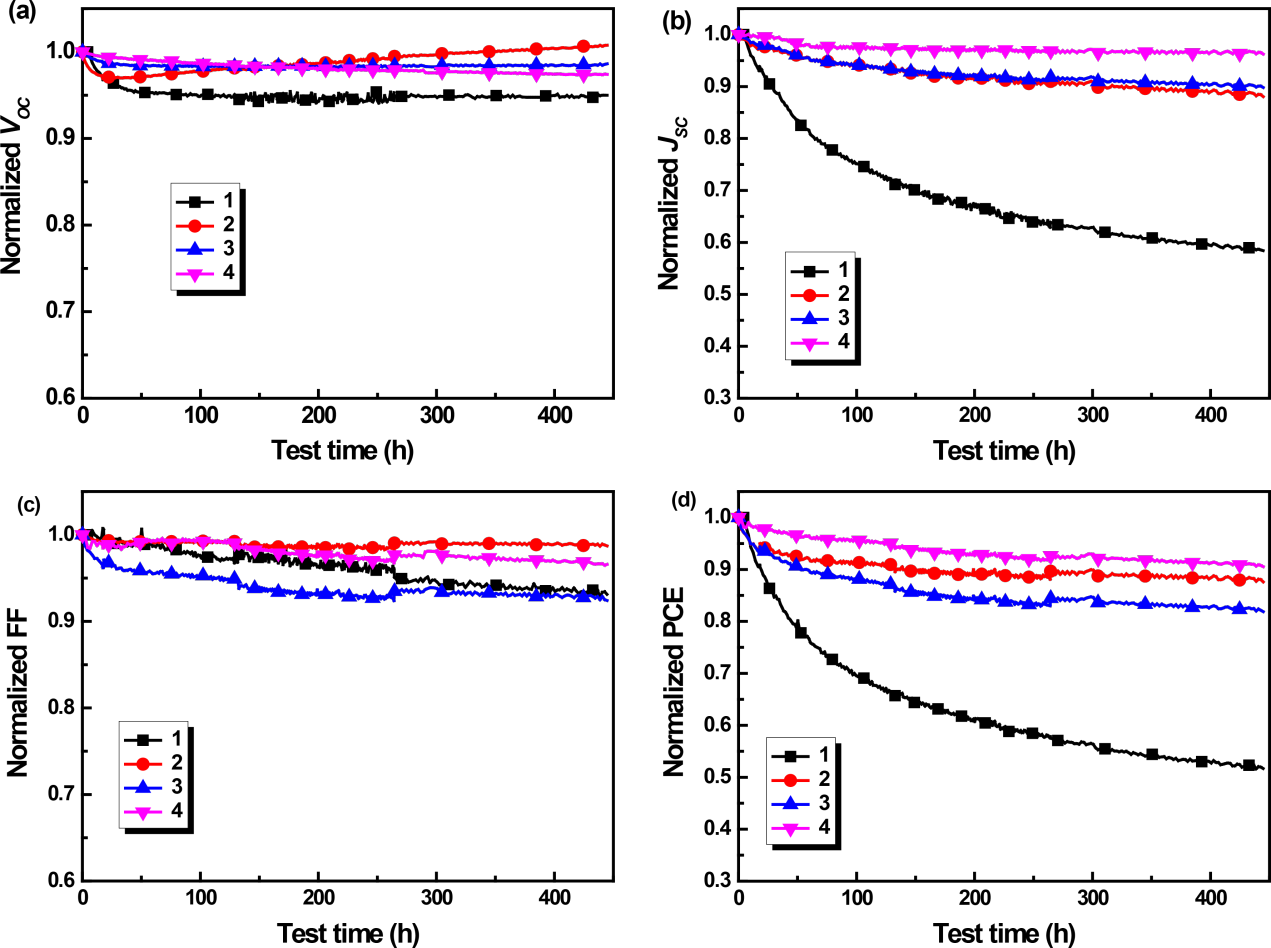
(a) *V*_OC_, (b) *J*_SC_, (c) FF, and (d) PCE decay of the COOP-*n*HT-TBDT:PC_61_BM solar cells. Note all these data are normalized to their initial value.

Finally, the long-term stability of these COOP-*n*HT-TBDT-based solar cells was tested. Devices for stability test were fabricated according to the optimized conditions described above, and these devices showed an initial performance similar to the best PCE as listed in Table 2 for each compound. Figure 6 presents the evolution of *V*_OC_, *J*_SC_, FF and PCE of un-encapsulated COOP-*n*HT-TBDT:PC_61_BM cells tested in N_2_ atmosphere under continuous illumination. To fully simulate the degradation behaviour of solar cells under working conditions, an external load to match the maximum power output point (mpp) was attached to each device, which was described in our previous report [38]. Similar to the previous report, the *V*_OC_ of the devices decreased only slightly during light illumination. However, the *J*_SC_ decreased to 42%, 12%, 10% and 4% of their initial value for cells based on **1**, **2**, **3** and **4**, respectively (Figure 6b). In addition, the FF of these four devices decreased very slowly (Figure 6c) during aging. Overall, the **4**-based devices showed the highest device stability with only 10% decay of its initial device performance, whereas the **1** showed the worst device stability, in which a decrease of 48% was measured. Surprisingly, **2** showed also higher device stability when compared to **3**, which was mainly due to a more stable FF. More nanomophology stability was supposed to be main reason for the higher stability of the larger molecules, since larger molecules have a higher energy barrier for diffusion. However, more experiments are still needed to further understand the stability improvement of the larger molecules.

## Conclusion

Four small A–π–D–π–A molecules with BDT core (COOP-*n*HT-TBDT, *n* = 1, 2, 3, 4) with regioregular oligo(3-hexylthiophene) π-bridges were synthesized and characterized. The length of the π-conjugation bridges has a significant impact on the optical, electrochemical properties and, in consequence, the device performance. With the elongation of the conjugated chain, broader absorption bands and narrower optical band gaps were observed for this type of compound, which would be beneficial for increasing *J*_SC_ in solar cell applications. A high *V*_OC_ of 0.9–1.0 V was achieved for the COOP-*n*HT-TBDT:PC_61_BM cells, owing to the low-lying HOMO levels of these compounds. The lengthening of the conjugated π-bridges improves the performance of the COOP-*n*HT-TBDT:PC_61_BM cells, and a maximum PCE of 5.62% with a *V*_OC_ of 0.93 V, *J*_SC_ of 9.60 mA·cm^−2^, and FF of 0.63 was achieved for the **4**:PC_61_BM-based device. In addition, improved device stability was also found for the larger molecules, which could be ascribed to the higher stability of the nanomorphology.

## Supporting Information

Supporting Information features experimental details about the synthesis of COOP-*n*HT-TBDT, the determination of molecular molar extinction coefficient of COOP-*n*HT-TBDT, the *J*–*V* curves and EQE spectra of organic solar cells based on COOP-*n*HT-TBDT at different blend ratios, a *J*–*V* comparison of devices based on **4**:PC_61_BM and **4**:PC_71_BM, UV–vis absorption spectra of COOP-*n*HT-TBDT:PC_61_BM blended films, as well as the NMR and MALDI–TOF MS spectra of COOP-*n*HT-TBDT.

File 1 Additional experimental data.
