# Supplementary material for: Effect of the π-conjugation length on the properties and photovoltaic performance of A–π–D–π–A type oligothiophenes with a 4,8-bis(thienyl)benzo[1,2-b:4,5-b′]dithiophene core
Source: Beilstein J Org Chem. 2016 Aug 10;12:1788–97. doi: 10.3762/bjoc.12.169 (PMC5082721; doi:10.3762/bjoc.12.169)
Supplement: File 1 — Additional experimental data. [file Beilstein_J_Org_Chem-12-1788-s001.pdf]

Supporting Information

for

Effect of the π-conjugation length on the properties and photovoltaic performance of A–π–D–π–A type oligothiophenes with a 4,8-bis(thienyl)-benzo[1,2-*b*:4,5-*b'*]dithiophene core

Ni Yin[1,2], Lilei Wang[1,3], Yi Lin[4], Jinduo Yi[1], Lingpeng Yan[1], Junyan Dou[1], Hai-Bo Yang[3], Xin Zhao*[2] and Chang-Qi Ma*[1]

Address: [1]Printable Electronics Research Center, Suzhou Institute of Nano-Tech and Nano-Bionics (SINANO), Chinese Academy of Sciences, 398 Ruo Shui Road, SEID SIP, Suzhou, Jiangsu, 215123, P. R. China; [2]College of Chemistry, Biology and Material Engineering, Suzhou University of Science and Technology, 1 Ke Rui Road, Suzhou, Jiangsu, 215009, P. R. China; [3]Department of Chemistry, Shanghai Key Laboratory of Green Chemistry and Chemical, East China Normal University, 3663 North Zhongshan Road, Shanghai 200062, P. R. China and [4]Department of Chemistry, Xi'an Jiaotong Liverpool University, 111 Ren Ai Road, Dushu Lake Higher Education Town, Suzhou, Jiangsu, 215123, P. R. China

Email: Xin Zhao* - zhaoxinsz@126.com; Chang-Qi Ma* – cqma2011@sinano.ac.cn

* Corresponding author

Additional experimental data

Experimental

Materials and reagents

Chemicals, including: 2-bromo-3-hexylthiophene (Puyang Huicheng Chemical Co. Ltd.), $Pd_2(dba)_3{\cdot}CHCl_3$ and HP*t*-$Bu_3{\cdot}BF_4$ (Sigma-Aldrich), cctyl cyanoacetate, piperidine, malononitrile, and β-alanine (Energy Chemical Co. Ltd.), 1,1'-[4,8-bis[5-(2-ethylhexyl)-2-thienyl]benzo[1,2-*b*:4,5-*b*']dithiophene-2,6-diyl]bis[1,1,1-trimethylstannane] (**16**, Derthon Optoelectronic Materials Co., Ltd) $PC_{61}BM$ (Solarmer Materials (Beijing) Inc.) and comment organic solvents (Sinopharm Chemical Reagent Co., Ltd and Chinasun Specialty Products Co., Ltd) were purchased from commercial sources. Anhydrous tetrahydrofuran (THF) and chloroform for device fabrication were purified according to a standard method [1]. 2-Bromo-3-hexylthiophene was distilled before use. Other chemicals were used as received. 5-Bromo-4-hexylthiophene-2-carbaldehyde (**9**) [2], 2-(4,4,5,5-tetramethyl-1,3,2-dioxaborolan-2-yl)-5-trimethylsilane-4-hexylthiophene (**6**) [3], and 5(4,4,5,5-tetramethyl-1,3,2-dioxaborolan-2-yl)-5'-(trimethylsilyl)-3,4'-dihexyl-2,2'-bithiophene (**8**) [4] were synthesized according to the literature. Synthesis procedures of the aldehyde terminated precursors (CHO-*n*HT-TBDT) and the final compounds (COOP-*n*HT-TBDT) are similar to the method reported in our previous paper [5]. Chemical structure characterization, organic solar cell fabrication and testing conditions, as well as the long-term stability testing for organic solar cells have been reported in our previous papers [5,6].

Measurements and characterization

Nuclear magnetic resonance (NMR) spectra were recorded using a Bruker Avance III 400 MHz and $CDCl_3$ as solvent. Chemical shifts are reported as δ values (ppm) with tetramethylsilane (TMS) as the internal standard. GC-MS was measured on Agilent GC-MS 5975C spectrometry. Matrix-assisted laser desorption ionization time-of-flight mass spectrometry (MALDI-TOF) spectra were recorded on a Brucker Autoflex Speed using *trans*-2-[3-(4-*tert*-butylphenyl)-2-methyl-2-propenylidene]malononitrile (DCTB) as the matrix. UV–vis absorption spectrum was recorded on a PerkinElmer Lambda 750. For UV–vis absorption spectrum measurement in solution, three solutions (around 10^{-3}·mol·L^{-1}) were prepared independently, each of which were further diluted to get three diluted solutions (with concentrations around 10^{-7} to 10^{-6} mol·L^{-1}) for UV–vis absorption measurements. The absorption spectra of the dilute solutions were recorded, and the data points of the absorbance at a certain wavelength vs concentration were then plotted. A good linear relationship was found for all these compounds, suggesting no obvious intermolecular interaction was found in such a concentration range. The molecular molar extinction coefficient (ε) was obtained from the slope of the best-fit line over the above mentioned data points according to the Beer–Lambert equation, $A = \varepsilon \cdot L \cdot c$. Thin solid films for UV–vis absorption measurements were prepared by spin coating a chloroform solution (8 mg·mL^{-1}) on quartz. Cyclic voltammetry (CV) was obtained in a tetrabutylammonium hexafluorophosphate (Bu_4NPF_6, 0.1 mol·L^{-1}) supported CH_2Cl_2 solution at room temperature using a RST3000 electrochemical workstation (Suzhou Risetech Instrument Co., Ltd) operated at a scanning rate of 100 mV·s^{-1}. A Pt wire (1.0 mm diameter)

embedded in Teflon column was used as the working electrode, and a Pt sheet and Ag/AgCl electrodes were served as the counter and reference electrodes, respectively. Ferrocene/ferrocenium was used as the internal reference to calibrate the redox potentials.

Fabrication and characterization of organic solar cells

The devices were fabricated with a traditional structure of glass/ITO/PEDOT:PSS (30 nm)/photoactive layer/LiF (1.5 nm)/Al (100 nm). The ITO-coated glass substrates were cleaned by ultrasonic treatment in detergent, deionized water, acetone, and isopropyl alcohol under ultrasonication for 30 min each, and dried by a nitrogen blow subsequently. After routine solvent cleaning, the ITO substrates were treated with UV ozone for 30 min and 30 nm of PEDOT:PSS (Clevios P VP AI 4083, filtered through 0.45 μm) was spin-coated at 3500 rpm. After transferred into an N_2-filled glovebox, the substrates were baked at 124 °C for 10 min. After that, the active layer was spin-coated from donor-acceptor blend chloroform solutions with different ratios. At last, LiF (1.5 nm) and Al (100 nm) were evaporated onto the active layer under vacuum (pressure below 1×10^{-4} Pa) through a shadow mask to form the cathodes. The effective area of the devices was 0.16 cm^2 or 0.09 cm^2, and both two types of devices provide similar performance. The active layer thickness was measured using an AlphaStep profilometer (Veeco, Dektak 150). The current density–voltage (*J*–*V*) characteristics were measured in a N_2-filled glove box using a Keithley 2400 source meter under an AM 1.5G filter (100 $mW \cdot cm^{-2}$) generated by white light of a tungsten halogen lamp, filtered by a Schott GG385 UV filter and a Hoya LB120 daylight filter. External quantum efficiencies (EQE)

were measured under simulated one sun operation condition using bias light from a 532 nm solid-state laser (Changchun New Industries, MGL-III-532). Light from a 150 W tungsten halogen lamp (Osram 64610) was used as probe light and modulated with a mechanical chopper before passing the monochromator (Zolix, Omni-λ300) to select the wavelength. The response was recorded as the voltage by an *I*–*V* converter (DNR-IV Convertor, Suzhou D&R Instruments), using a lock-in amplifier (Stanford Research Systems SR 830). A calibrated Si cell was used as reference. The device for EQE measurement was kept behind a quartz window in a nitrogen filled container.

Long-term stability test of the organic solar cells

The long-term stability of un-encapsulated devices was tested using a thin film solar cell decay testing system (PVLT-G801M, Suzhou D&R Instruments) under the testing condition that is in accordance with ISOS-L-1. In detail, the un-encapsulated devices were put inside a glove box (H_2O < 10 ppm, O_2 < 10 ppm), and continuously illuminated with LED white light (color temperature, 6000 K). The illumination light intensity was initially set as the output device short circuit current equals to J_{SC} measured under standard conditions. The illumination light intensity was monitored by a photodiode (Hamamtsu S1336-8BQ). Maximum power outputs (mpp) of these devices were checked periodically according to the *J*–*V* measurement, and the devices were attached with an external load at the maximum power output point under illumination. The device temperature was monitored to be around 35–40 °C during the testing.

Synthesis of materials

3,4'-dihexyl-5'-(trimethylsilyl)-2,2'-bithiophene-5-carbaldehyde (**10**):

A solution of **9** (5.60 g, 15.3 mmol), **6** (3.00 g, 10.9 mmol), $Pd_2(dba)_3{\cdot}CHCl_3$ (226 mg, 21.8 μmol) and HP*t*-$Bu_3{\cdot}BF_4$ (127 mg, 43.6 μmol) in THF (100 mL) was degassed by bubbling N_2 for 30 min. A N_2-degrassed K_2CO_3 solution (1M, 30 mL, 30 mmol) was added and the resulting mixture was vigorously stirred at room temperature overnight under N_2 atmosphere. The solution was extracted with ethyl ether after THF was taken off. The organic layer was dried over Na_2SO_4 and the solvent was removed under vacuum. The crude product was purified by flash chromatography (SiO_2, *n*-hexane/CH_2Cl_2 = 2:1) to give a yellow oil (4.30 g, 90% yield). ^{1}H NMR ($CDCl_3$, 400 MHz) δ ppm: 9.81 (s, 1H), 7.57 (s, 1H), 7.20 (s, 1H), 2.80 (t, J =8.0 Hz, 2H), 2.65 (t, J =7.6 Hz, 2H), 1.70~1.55 (m, 4H), 1.42~1.31 (m, 12H), 0.92~0.88 (m, 6H), 0.37 (s, 9H). ^{13}C NMR (100 MHz, $CDCl_3$) δ ppm: 182.66, 151.15, 141.88, 140.17, 140.11, 139.11, 138.71, 136.20, 130.81, 31.84, 31.78, 31.69, 31.43, 30.36, 29.45, 29.43, 29.21, 14.17, 0.39.

3,4'-dihexyl-5'-iodo-2,2'-bithiophene-5-carbaldehyde (**11**):

To a solution of **10** (1.20 g, 2.76 mmol) in 12 mL THF was added dropwise a solution of iodine monochloride (6.1 mL, 0.9 M in THF, 5.49 mmol) at −78 °C. Then the reaction mixture was stirred for one hour and stirred at room temperature for another one hour. After that, the reaction was then quenched by $Na_2S_2O_5$ (1 M, 10 mL, 10 mmol). The organic layer was separated and the aqueous layer was extracted with CH_2Cl_2. The combined organic extract was washed with brine, then dried over Na_2SO_4. The solvent

was removed by rotary evaporator and the residue was purified by flash chromatography (SiO_2, *n*-hexane/CH_2Cl_2 = 2:1) to give a yellow oil (1.01 g, 75% yield). ^1H NMR ($CDCl_3$, 400 MHz) δ ppm: 9.83 (s, 1H), 7.57 (s, 1H), 6.91 (s, 1H), 2.75 (t, *J* =7.6 Hz, 2H), 2.65 (t, *J* = 8.0 Hz, 2H), 1.69~1.56 (m, 4H), 1.43~1.27 (m, 12H), 0.92~0.87 (m, 6H). ^{13}C NMR (100 MHz, $CDCl_3$) δ ppm: 182.56, 148.14, 140.65, 140.56, 140.49, 139.56, 138.82, 127.97, 76.93, 32.32, 31.67, 31.65, 30.31, 29.96, 29.34, 29.16, 28.93, 22.66, 66.65 14.16, 14.14.

3,4',4''-trihexyl-5''-(trimethylsilyl)-2,2':5',2''-terthiophene-5-carbaldehyde (**12**):

A solution of **11** (3.88 g, 7.95 mmol), **6** (4.37 g, 11.9 mmol), $Pd_2(dba)_3{\cdot}CHCl_3$ (165 mg, 0.159 mmol) and HP*t*-$Bu_3{\cdot}BF_4$ (92 mg, 0.318 mmol) in THF (80 mL) was degassed by bubbling N_2 for 30 min. A N_2-degrassed K_2CO_3 solution (1M, 20 mL, 20 mmol) was added and the resulting mixture was added and the resulting solution was vigorously stirred at room temperature overnight under N_2 atmosphere. The solution was extracted with ethyl ether after THF was taken off. The organic layer was dried over Na_2SO_4 and the solvent was removed in vacuo. The crude product was purified by flash chromatography (SiO_2, *n*-hexane/CH_2Cl_2 = 2:1) to give **12** (3.90 g, 82% yield) as an orange oil. ^1H NMR ($CDCl_3$, 400 MHz) δ ppm: 9.81 (s, 1H), 7.58 (s, 1H), 7.11 (s, 1H), 7.09 (s, 1H), 2.82 (t, *J* = 7.6 Hz, 2H), 2.77 (t, *J* = 8.0 Hz, 2H), 2.66 (t, *J* = 7.6 Hz, 2H), 1.72~1.61 (m, 6H), 1.43~1.31 (m, 18H), 0.92~0.88 (m, 9H), 0.37 (s, 9H). ^{13}C NMR (100 MHz, $CDCl_3$) δ ppm: 182.55, 150.93, 143.86, 141.53, 141.42, 140.13, 139.96, 139.89, 139.83, 139.17, 139.13, 139.02, 134.87, 134.10, 133.51, 133.47, 132.27, 130.41, 130.35, 129.53, 127.69, 120.70, 31.78, 31.70,

31.64, 31.45, 30.53, 30.47, 30.42, 30.25, 29.42, 29.27, 29.21, 29.18, 29.16, 29.02, 22.64, 22.62, 14.11, 1.96, 1.36, 0.40. MS (MALDI-TOF): calcd. for $C_{34}H_{52}OS_3Si$ $[M]^+$, 600.29 ; found,600.32.

3,4',4''-trihexyl-5''-iodo-2,2':5',2''-terthiophene-5-carbaldehyde (**13**):

To a solution of **12** (0.65 g, 1.08 mmol) in 5 ml THF was added dropwise a solution of iodine monochloride (2.4 mL, 0.9 M in THF, 2.16 mmol) at −78 °C. Then the reaction mixture was stirred for one hour and stirred at room temperature for another one hour. After that, the reaction was then quenched by $Na_2S_2O_5$ (1 M, 5 mL, 5 mmol). The organic layer was separated and the aqueous layer was extracted with $CHCl_3$. The combined organic extract was washed with brine, then dried over Na_2SO_4. The solvent was removed by rotary evaporator and the residue was purified by flash chromatography (SiO_2, *n*-hexane/CH_2Cl_2 = 2:1) to give **13** (0.49 g, 70% yield) as an orange oil. ^1H NMR ($CDCl_3$, 400 MHz) δ ppm: 9.81 (s, 1H), 7.56 (s, 1H), 7.08 (s, 1H), 6.79 (s, 1H), 2.79 (t, *J* = 8.0 Hz, 2H), 2.71 (t, *J* = 7.6 Hz, 2H), 2.54 (t, *J* = 8.0 Hz, 2H), 1.71~1.56 (m, 6H), 1.41~1.25 (m, 18H), 0.91~0.87 (m, 9H). ^{13}C NMR (100 MHz, $CDCl_3$) δ ppm: 182.42, 147.25, 140.96, 140.23, 140.11, 139.77, 138.96, 132.82, 132.37, 130.18, 126.78, 74.76, 32.28, 31.57, 30.43, 30.18, 29.91, 29.36, 29.17, 29.10, 29.09, 28.86, 22.56, 14.04. MS (MALDI-TOF): calcd. for $C_{31}H_{43}IOS_3$ [M]+, 654.15; found, 654.13.

3,4',4'',4'''-tetrahexyl-5'''-(trimethylsilyl)-2,2':5',2'':5'',2'''-quathiophene-5-carbaldehyde (**14**):

A solution of **11** (1.70 g, 3.48 mmol), **8** (2.41 g, 4.53 mmol), $Pd_2(dba)_3{\cdot}CHCl_3$ (72.0 mg,

69.6 μmol) and $HP^tBu_3 \cdot BF_4$ (41.0 mg, 0.14 mmol) in THF (30 mL) was degassed by bubbling N_2 for 30 min. A N_2-degrassed K_2CO_3 solution (1M, 10 mL, 10 mmol) was added and the resulting mixture was vigorously stirred at room temperature for 48 h under N_2 atmosphere. The solution was extracted with $CHCl_3$ after THF was taken off. The organic layer was dried over Na_2SO_4 and the solvent was removed in vacuum. The crude product was purified by flash chromatography (SiO_2, *n*-hexane/$CHCl_3$ = 2:1) to give an orange oil (2.30 g, 86% yield). ^1H NMR ($CDCl_3$, 400 MHz) δ ppm: 9.82 (s, 1H), 7.58 (s, 1H), 7.12 (s, 1H), 7.08 (s, 1H), 6.99 (s, 1H), 2.84-2.75 (m, 6H), 2.65 (t, *J* = 7.2 Hz, 2H), 1.72~1.59 (m, 8H), 1.43~1.32 (m, 24H), 0.92~0.88 (m, 12H), 0.37 (s, 9H). ^{13}C NMR (100 MHz, $CDCl_3$) δ ppm: 182.53, 150.87, 143.75, 141.38, 140.21, 140.07, 140.02, 139.96, 139.74, 139.63, 139.46, 139.15, 135.28, 133.48, 133.07, 132.74, 132.40, 132.32, 131.80, 130.51, 129.19, 129.05, 129.02, 127.34, 120.27, 31.80, 31.72, 31.49, 30.60, 30.54, 30.47, 30.26, 29.23, 14.13, 1.97, 1.37, 0.43. MS (MALDI-TOF): calcd. for $[M]^+$, 766.38; found, 766.40.

3,4',4'',4'''-tetrahexyl-5'''-iodo-2,2':5',2'':5'',2'''-quathiophene-5-carbaldehyde (**15**):

To a solution of **14** (1.40 g, 1.83 mmol) in 10 mL THF was added dropwise a solution of iodine monochloride (4.1 mL, 0.9 M in THF, 3.65 mmol) at −78 °C. Then the reaction mixture was stirred for one hour and stirred at room temperature for another one hour. After that, the reaction was then quenched by $Na_2S_2O_5$ (1 M, 5 mL, 5 mmol). The organic layer was separated and the aqueous layer was extracted with ethyl ether. The combined organic extract was washed with brine, then dried over Na_2SO_4. The solvent was removed by rotary evaporator and the residue was purified by flash chromatography (SiO_2,

n-hexane/$CHCl_3$ = 2:1) to give an orange oil (1.08 g, 72% yield). ^{1}H NMR ($CDCl_3$, 400 MHz) δ ppm: 9.82 (s, 1H), 7.58 (s, 1H), 7.12 (s, 1H), 6.98 (s, 1H), 6.79 (s, 1H), 2.84~2.70 (m, 6H), 2.55 (t, *J* = 7.2 Hz, 2H), 1.73~1.57 (m, 8H), 1.43~1.27 (m, 24H), 0.92~0.87 (m, 12H). ^{13}C NMR (100 MHz, $CDCl_3$) δ ppm: 182.52, 147.72, 141.20, 140.30, 140.27, 140.23, 140.07, 139.11, 133.37, 132.76, 132.62, 130.80, 130.48, 128.94, 126.54, 74.28, 32.37, 31.68, 31.65, 30.58, 30.47, 30.26, 29.99, 29.46, 29.37, 29.31, 29.23, 29.18, 28.95, 22.65, 14.13. MS (MALDI-TOF): calcd. for $C_{41}H_{57}IOS_4$ $[M]^+$, 820.23; found, 820.21.

4,8-bis[5-(2-ethylhexyl)-2-thienyl]-2,6-bis[3,3'-dihexyl-5'-aldehyde-5,2'-bithiophen-2-yl]benzo[1,2-*b*:4,5-*b*']dithiophene (**18**)

A mixture of **11** (470 mg, 0.962 mmol), **16** (396 mg, 0.437 mmol), and $Pd(PPh_3)_4$ (50 mg, 43.7 µmol) was stirred at 80 °C in DMF (4 mL) for 16 h. After cooled to room temperature, the reaction mixture was added dropwise to anhydrous methanol (15 mL) and stirred for 0.5 hour at room temperature. A dark red solid was obtained by filtering through a Büchner funnel. The residue was purified by flash chromatography (SiO_2, *n*-hexane/CH_2Cl_2 = 1:1) to give **17** (465 mg, 82 %) as dark red solid. ^{1}H NMR ($CDCl_3$, 400 MHz) δ ppm: 9.83 (s, 2H), 7.70 (s, 2H), 7.59 (s, 2H), 7.35 (d, *J* = 3.6 Hz, 2H), 7.14 (s, 2H), 6.91 (d, *J* = 3.6 Hz, 2H), 2.89~2.80 (m, 12H), 1.73~1.65 (m, 10H), 1.40~1.25 (m, 40H), 0.97~0.87 (m, 24H). ^{13}C NMR (100 MHz, $CDCl_3$) δ ppm: 182.56, 146.11, 142.48, 141.71, 140.97, 140.57, 140.30, 139.17, 139.00, 136.96, 136.79, 136.46, 134.03, 133.02, 131.76, 130.64, 127.85, 125.52, 123.55, 122.12, 41.50, 34.31, 32.55, 31.89, 31.64, 31.61, 30.55, 30.22, 29.52, 29.41, 29.24, 29.10, 28.94, 25.73, 23.05, 22.64, 22.59, 14.17, 14.11, 14.08, 10.92. MS

(MALDI-TOF): calcd. for $C_{76}H_{98}O_2S_8$ $[M]^+$, 1298.53; found 1298.46.

4,8bis[5(2-ethylhexyl)2-thienyl]-2,6-bis[3,3',3''trihexyl-5''-aldehyde-5,2':5',2''terthiophen2-yl]benzo[1,2-*b*:4,5-*b*']dithiophene (**19**):

A mixture of **13** (321 mg, 0.491 mmol), **16** (202 mg, 0.223 mmol), and $Pd(PPh_3)_4$ (26 mg, 22.3 μmol) was stirred at 80 °C in DMF (3 mL) for 16 h. After cooled to room temperature, the reaction mixture was added dropwise to anhydrous methanol (10 mL) and stirred for 0.5 h at room temperature. A dark red solid was obtained by filtering through a Büchner funnel. The residue was purified by flash chromatography (SiO_2, *n*-hexane/CH_2Cl_2 = 1:1) to give **19** (220 mg, 60 %) as a dark red solid. ^1H NMR ($CDCl_3$, 400 MHz) δ ppm: 9.83 (s, 2H), 7.68 (s, 2H), 7.59 (s, 2H), 7.35 (d, J = 3.6 Hz, 2H), 7.13 (s, 2H), 7.03 (s, 2H), 6.91 (d, J = 3.6 Hz, 2H), 2.89~2.78 (m, 16H), 1.74~1.65 (m, 14H), 1.49~1.31 (m, 52H), 0.98~0.88 (m, 30H). ^{13}C NMR (100 MHz, $CDCl_3$) δ ppm: 182.51, 145.93, 141.49, 141.17, 140.40, 140.31, 140.07, 139.05, 136.92, 136.72, 134.50, 132.72, 131.39, 130.47, 129.31, 127.26, 125.44, 123.46, 121.61, 41.47, 34.28, 32.53, 31.64, 30.57, 30.39, 30.21, 29.57, 29.41, 29.26, 29.12, 28.92, 25.71, 23.02, 22.63, 22.58, 14.15, 14.07, 10.89. MS (MALDI-TOF): calcd. for $C_{96}H_{126}O_2S_{10}$ $[M]^+$, 1630.69; found 1631.65.

4,8bis[5-(2-ethylhexyl)-2-thienyl]-2,6-bis[3,3',3'',3'''tetrahexyl-5'''aldehyde-5,2':5',2'':5'',2'''-quathiophen2-yl]benzo[1,2-*b*:4,5-*b*']dithiophene (**20**):

A mixture of **15** (500 mg, 0.609 mmol), **16** (250 mg, 0.277 mmol), and $Pd(PPh_3)_4$ (32 mg, 27.7 μmol) was stirred at 80 °C in DMF (3 mL) for 16 h. After cooled to room temperature,

the reaction mixture was added dropwise to anhydrous methanol (15 mL) and stirred for 0.5 h at room temperature. A dark red solid was obtained by filtering through a Büchner funnel. The residue was purified by flash chromatography (SiO_2, *n*-hexane/$CHCl_3$ = 2:1) to give **20** (398 mg, 81 %) as a dark red solid. ^{1}H NMR ($CDCl_3$, 400 MHz) δ ppm: 9.83 (s, 2H), 7.68 (s, 2H), 7.59 (s, 2H), 7.36 (d, *J* = 3.6 Hz, 2H), 7.13 (s, 2H), 7.01 (d, *J* = 1.6 Hz, 4H), 6.92 (d, *J* = 3.6 Hz, 2H), 2.89~2.79 (m, 20H), 1.74~1.66 (m, 18H), 1.46~1.28 (m, 64H), 1.00~0.92 (m, 36H). ^{13}C NMR (100 MHz, $CDCl_3$) δ ppm: 182.53, 145.91, 141.44, 141.27, 140.30, 140.26, 140.24, 140.05, 139.11, 139.02, 137.01, 136.94, 136.85, 134.97, 133.25, 132.91, 132.56, 131.08, 130.95, 130.52, 129.16, 128.98, 127.78, 125.46, 123.31, 121.49, 41.50, 34.32, 32.56, 31.68, 31.64, 31.60, 30.46, 30.25, 29.62, 29.22, 29.16, 28.96, 25.74, 23.06, 22.67, 22.64, 22.62, 14.19, 14.12, 10.92. MS (MALDI-TOF): calcd. for $C_{116}H_{154}O_2S_{12}$ $[M]^+$, 1962.86; found 1962.73.

4,8-bis[5-(2-ethylhexyl)-2-thienyl]2,6-bis[3,3'-dihexyl-5'(2-cyano-3octyloxy-3oxo-1-propenyl)- 5,2'-bithiophen-2-yl]benzo[1,2-*b*:4,5-*b*']dithiophene (**2**):

Octyl cyanoacetate (154 mg, 0.780 mmol) was added to a solution of **18** (390 mg, 0.300 mmol) and piperidine (0.1 mL) in dry $CHCl_3$ (80 mL) and then the solution was stirred for 24 h under N_2 at 60 °C. Water was added and the reaction mixture was extracted with $CHCl_3$, the combined extracts were washed three times with water and then dried ($MgSO_4$). The solvent was evaporated under reduced pressure and the crude product further purified through column chromatography (SiO_2, *n*-hexane/$CHCl_3$ = 3:2) to afford a dark powder (0.434 g, 87%). ^{1}H NMR ($CDCl_3$, 400 MHz) δ ppm: 8.20 (s, 2H), 7.71

(s, 2H), 7.58 (s, 2H), 7.36 (d, J = 3.6 Hz, 2H), 7.21 (s, 2H), 6.92 (d, J = 3.6 Hz, 2H), 4.29 (t, J = 6.8 Hz, 4H), 2.89~2.81 (m, 12H), 1.79~1.63 (m, 14H), 1.47~1.26 (m, 60H), 0.97~0.87 (m, 30H). ^{13}C NMR (100 MHz, $CDCl_3$) δ ppm: 163.15, 146.11, 146.03, 141.89, 141.66, 140.90, 140.67, 139.18, 137.00, 136.74, 136.45, 133.68, 133.52, 132.98, 131.04, 127.88, 125.55, 123.58, 122.14, 116.03, 97.77, 66.59, 41.49, 34.31, 32.55, 31.79, 31.63, 31.59, 30.58, 30.09, 29.57, 29.19, 29.11, 28.94, 28.58, 30.09, 29.57, 29.19, 29.11, 28.94, 28.58, 25.82, 25.73, 23.04, 22.65, 22.59, 14.17, 14.10, 14.08, 10.91. MS (MALDI-TOF): calcd. for $C_{98}H_{132}N_2O_4S_8$ $[M]^+$, 1656.79; found 1656.76.

4,8-bis[5-(2-ethylhexyl)-2-thienyl]-2,6-bis[3,3',3''-trihexyl-5''-(2-cyano-3-octyloxy-3-oxo-1-propenyl)- 5,2':5',2''-terthiophen-2-yl]benzo[1,2-*b*:4,5-*b*']dithiophene (**3**):

Octyl cyanoacetate (51 mg, 0.257 mmol) was added to a solution of **19** (140 mg, 0.0858 mmol) and piperidine (0.1 mL) in dry $CHCl_3$ (40 mL) and then the solution was stirred for 24 h under N_2 at 60 °C. Water was added and the reaction mixture was extracted with $CHCl_3$, the combined extracts were washed three times with water and then dried (Na_2SO_4). The solvent was evaporated under reduced pressure and the crude product further purified through column chromatography (SiO_2, *n*-hexane/$CHCl_3$ = 1:1) to afford a dark powder (0.136 g, 80%). ^{1}H NMR ($CDCl_3$, 400 MHz) δ ppm: 8.20 (s, 2H), 7.68 (s, 2H), 7.57 (s, 2H), 7.36 (d, J = 3.2 Hz, 2H), 7.19 (s, 2H), 7.04 (s, 2H), 6.92 (d, J = 3.6 Hz, 2H), 4.29 (t, J = 6.8 Hz, 4H), 2.89~2.78 (m, 16H), 1.79~1.63 (m, 18H), 1.47~1.26 (m, 72H), 0.98~0.88 (m, 36H). ^{13}C NMR (100 MHz, $CDCl_3$) δ ppm: 163.18, 146.01, 145.94, 141.90,141.53, 140.97, 140.58, 140.42, 139.04, 136.92, 136.71, 134.45, 133.45, 133.25,

132.77, 132.36, 131.50, 130.87, 129.37, 127.77, 125.45, 123.35, 121.64, 116.04, 97.54, 66.54, 41.47, 34.28, 32.53, 31.59, 30.55, 30.43, 29.58, 29.29, 29.26, 29.20, 29.17, 29.13, 28.92, 28.56, 25.79, 25.17, 23.02, 22.63, 22.59, 14.15, 14.07, 10.89. MS (MALDI-TOF): calcd. for $C_{118}H_{160}N_2O_4S_{10}$ $[M]^+$, 1988.96; found 1989.47.

4,8-bis[5-(2-ethylhexyl)-2-thienyl]-2,6-bis[3,3',3'',3'''-tetrahexyl-5'''-(2-cyano-3-octyloxy-3-oxo-1-propenyl)-5,2':5',2'':5'',2'''-quathiophen-2-yl]benzo[1,2-*b*:4,5-*b*']dithiophene (**4**): Octyl cyanoacetate (94 mg, 0.489 mmol) was added to a solution of compound **20** (385 mg, 0.196 mmol) and piperidine (0.1 mL) in dry $CHCl_3$ (80 mL) and then the solution was stirred for 24 h under N_2 at 60 °C. Water was added and the reaction mixture was extracted with $CHCl_3$, the combined extracts were washed three times with water and then dried (Na_2SO_4). The solvent was evaporated under reduced pressure and the crude product further purified through column chromatography (SiO_2, *n*-hexane/$CHCl_3$ = 1:1) to afford a dark brown solid (0.366 g, 80%). ^{1}H NMR ($CDCl_3$, 400 MHz) δ ppm: 8.20 (s, 2H), 7.68 (s, 2H), 7.57 (s, 2H), 7.32 (d, *J* = 3.2 Hz, 2H), 7.18 (s, 2H), 7.01 (s, 4H), 6.90 (d, *J* = 3.6 Hz, 2H), 4.29 (t, *J* = 6.8 Hz, 4H), 2.89~2.78 (m, 20H), 1.79~1.65 (m, 22H), 1.49~1.26 (m, 84H), 0.98~0.88 (m, 42H). ^{13}C NMR (100 MHz, $CDCl_3$) δ ppm: 163.22, 146.04, 145.91, 142.00, 141.02, 140.41, 140.30, 139.02, 137.01, 136.94, 136.85, 134.95, 133.44, 133.19, 132.75, 132.19, 131.20, 130.99, 130.93, 129.21, 129.00, 127.78, 125.46, 123.31, 121.50, 116.08, 97.51, 66.56, 41.50, 34.31, 32.56, 31.79, 31.66, 31.62, 30.60, 30.49, 30.46, 30.12, 29.62, 29.43, 29.33, 29.30, 29.27, 29.20, 29.17, 28.95, 28.59, 25.82, 25.74, 23.05, 22.66, 14.18, 14.10, 10.92. MS (MALDI-TOF): calcd. for $C_{138}H_{188}N_2O_4S_{12}$ $[M]^+$, 2321.12; found

2322.01.

References

[1]. Armarego, W. L. F.; Perrin, D. D., Purification of Laboratory Chemicals. 4 ed.; Butterwirth-Heinemann: Oxford, 1999.

[2]. Zhang, J.; Wu, G.; He, C.; Deng, D.; Li, Y., *J. Mater. Chem.* **2011**, 21, 3768-3774.

[3]. Li, J.-C.; Lee, S.-H.; Hahn, Y.-B.; Kim, K.-J.; Zong, K.; Lee, Y.-S., *Synth. Met.* **2008**, 158, 150-156.

[4]. Weidelener, M.; Mishra, A.; Nattestad, A.; Powar, S.; Mozer, A. J.; Mena-Osteritz, E.; Cheng, Y.-B.; Bach, U.; Bauerle, P., *J. Mater. Chem.* **2012**, 22, 7366-7379.

[5]. Yin, N.; Wang, L.; Ma, Y.; Lin, Y.; Wu, J.; Luo, Q.; Yang, H.-B.; Ma, C.-Q.; Zhao, X., *Dyes Pigments* **2015**, 120, 299-306.

[6] Wu, N.; Luo, Q.; Bao, Z.; Lin, J.; Li, Y.-Q.; Ma, C.-Q., *Sol. Energy Mater. Sol. Cells* **2015**, 141, 248-259.

UV-Vis absorption and excition coefficient measurement

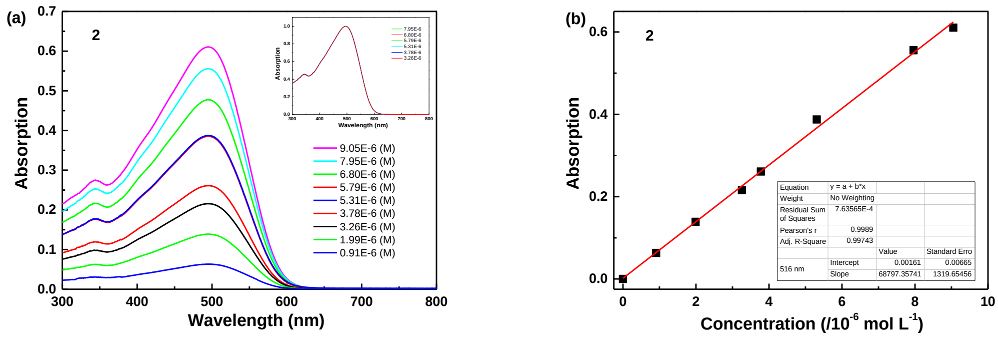

Figure S1: (a) UV−vis absorption spectra of **2** at different concentrations; (b) The fitting line of the data points of the absorbance at a certain wavelength vs. concentration.

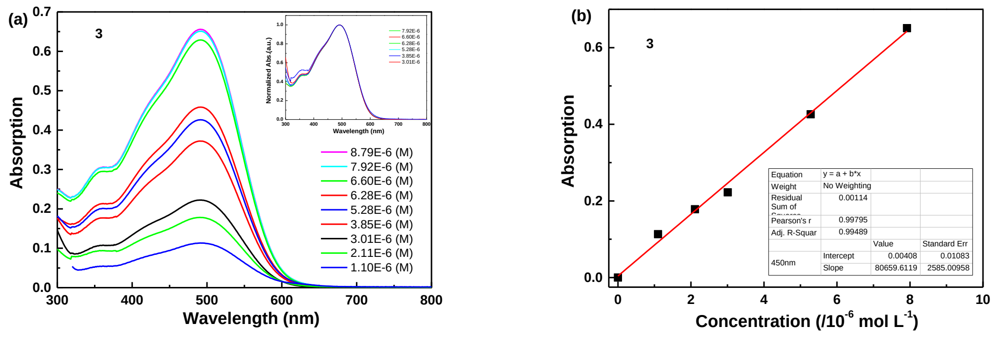

Figure S2: (a) UV−vis absorption spectra of **3** at different concentrations; (b) The fitting line of the data points of the absorbance at a certain wavelength vs. concentration.

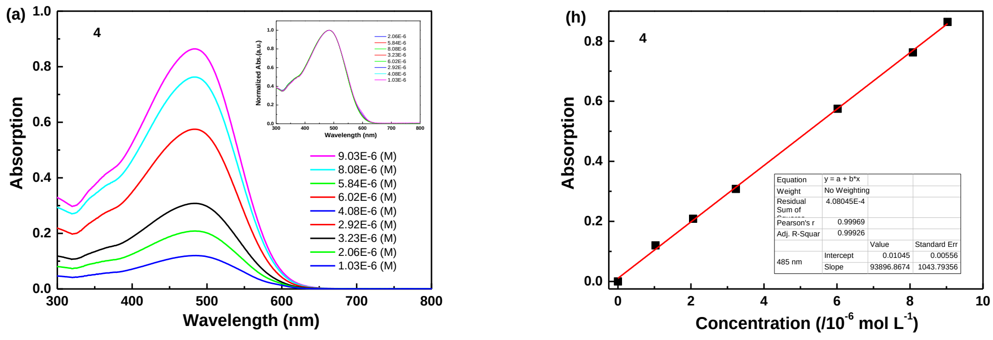

Figure S3: (a) UV−vis absorption spectra of **4** at different concentrations; (b) The fitting line of the data points of the absorbance at a certain wavelength vs. concentration.

J–*V* curves of organic solar cells at different blended ratio

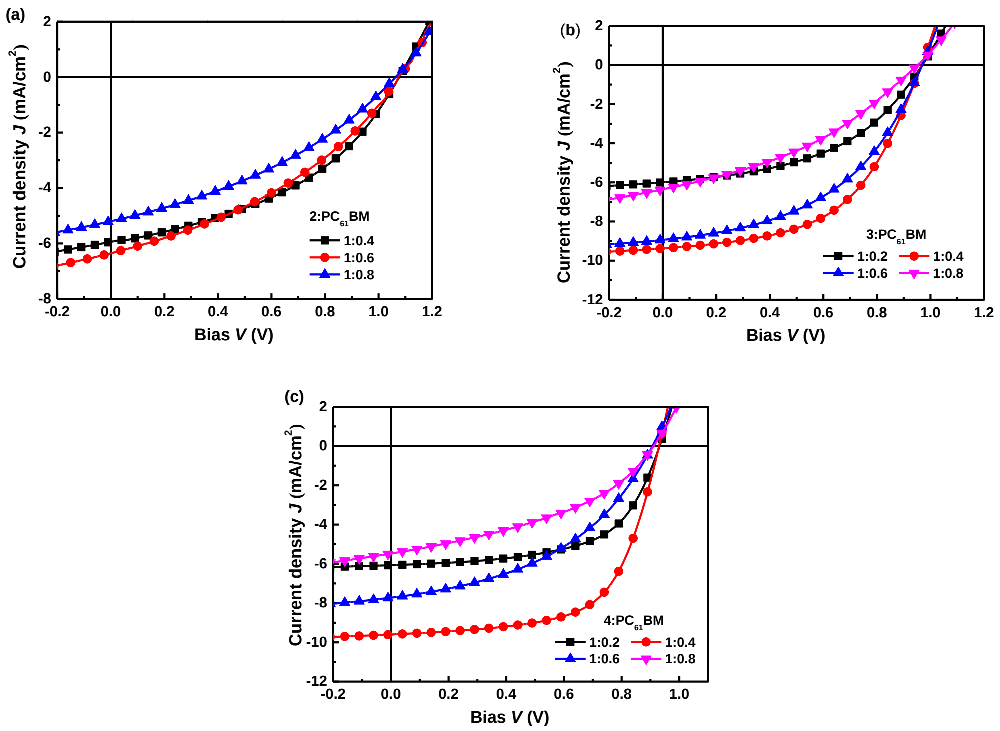

Figure S4: Current density (*J*)–voltage (*V*) curves of (a) **2**:PC_{61}BM; (b) **3**:PC_{61}BM; (c) **4**:PC_{61}BM BHJ solar cells at different D/A ratios.

EQE spectra of organic solar cells at different blended ratio

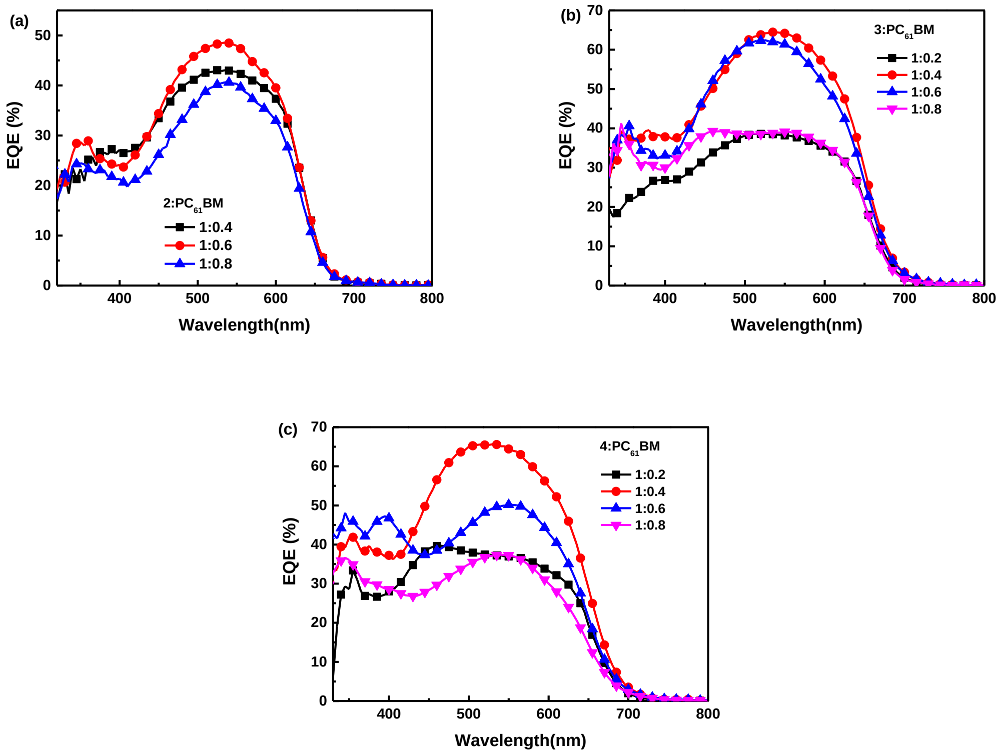

Figure S5: EQE curves of BHJ solar cells incorporating (a) **2**:PC$_{61}$BM, (b) **3**:PC$_{61}$BM and (c) **4**:PC$_{61}$BM blends of various weight ratios.

J–*V* curves comparison of 4:PC$_{61}$BM and 4:PC$_{71}$BM-based devices

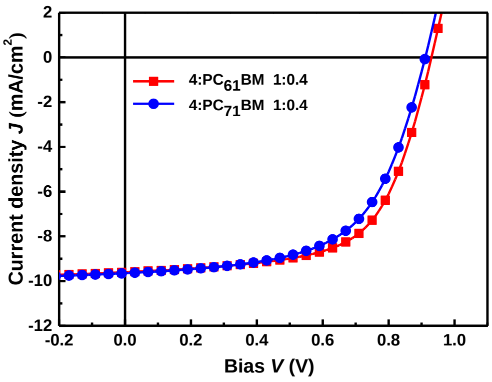

Figure S6: Current density (*J*)–voltage (*V*) curves of devices based on **4** and PC$_{61}$BM or PC$_{71}$BM at 1:0.4 (w/w).

UV–vis absorption spectra of COOP-*n*HT-TBDT:$PC_{61}BM$ blended films

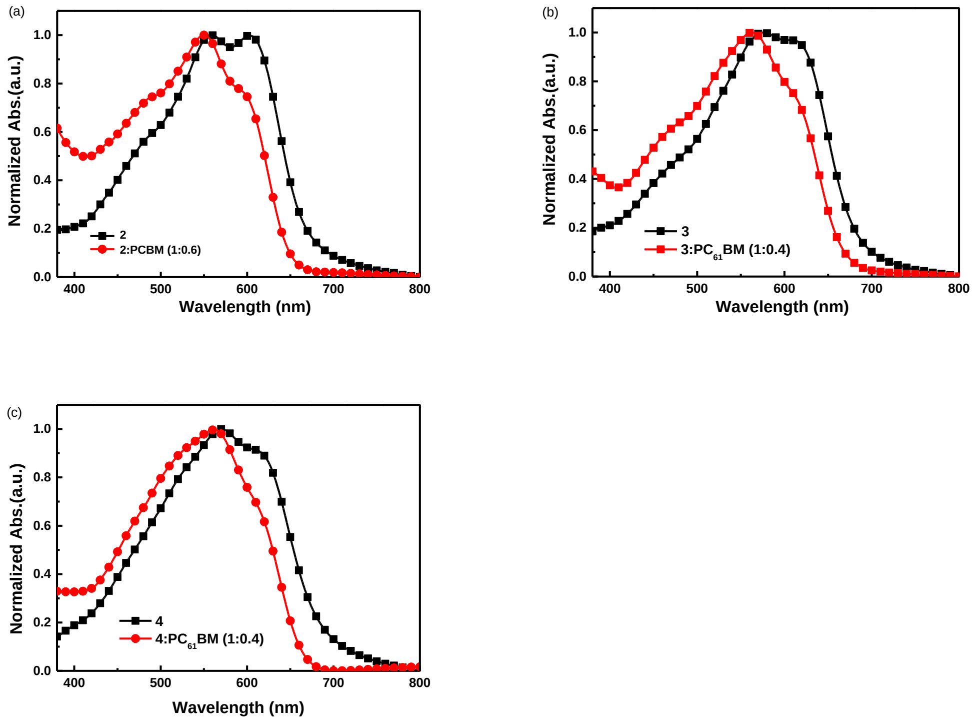

Figure S7: (a) Absorption spectra of **2** and **2**:$PC_{61}BM$ (1:0.6, w/w), (b) **3** and **3**:$PC_{61}BM$ (1:0.4, w/w) and (c) **4** and **4**:$PC_{61}BM$ (1:0.4, w/w) spin-coated from $CHCl_3$ onto glass substrates.

NMR spectra

(a)

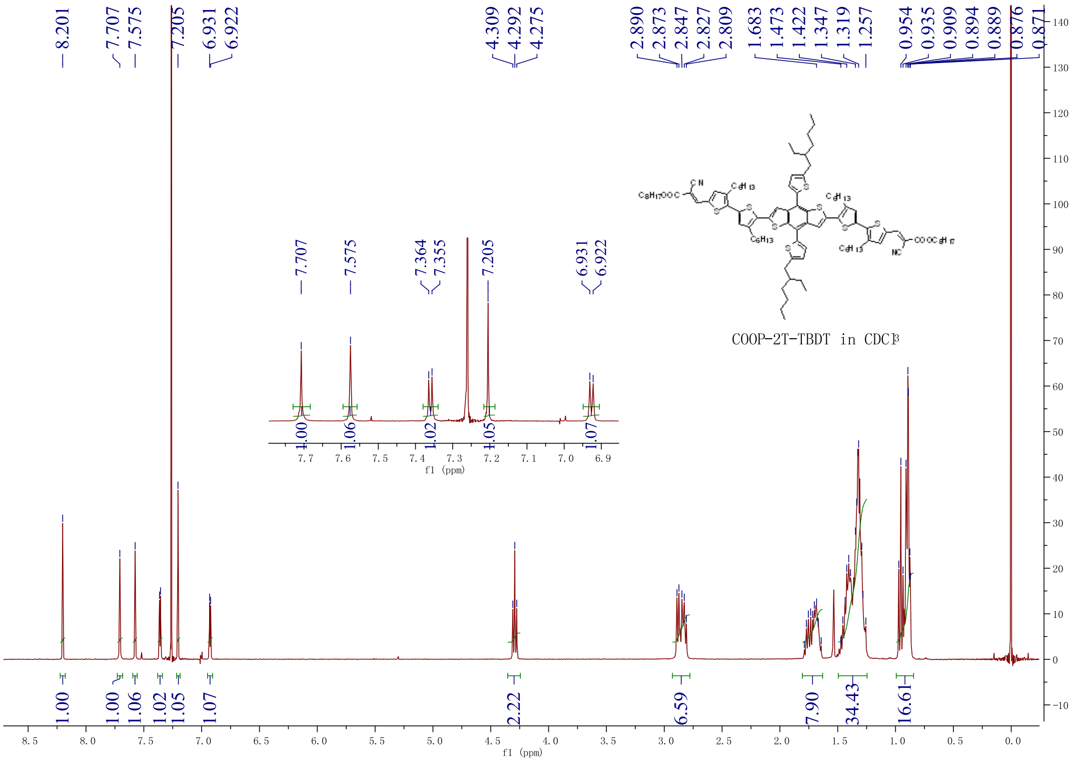

(b)

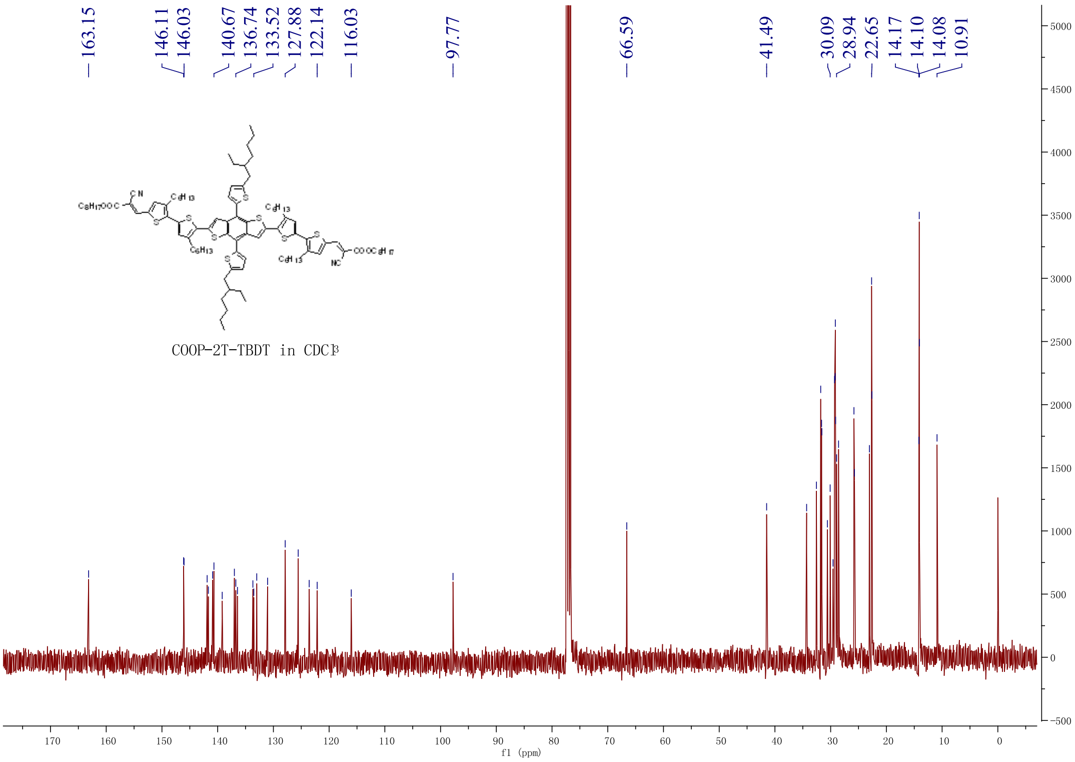

Figure S8: (a) ^1H and (b) ^{13}C NMR spectra of COOP-2T-TBDT ($CDCl_3$, 400MHz)

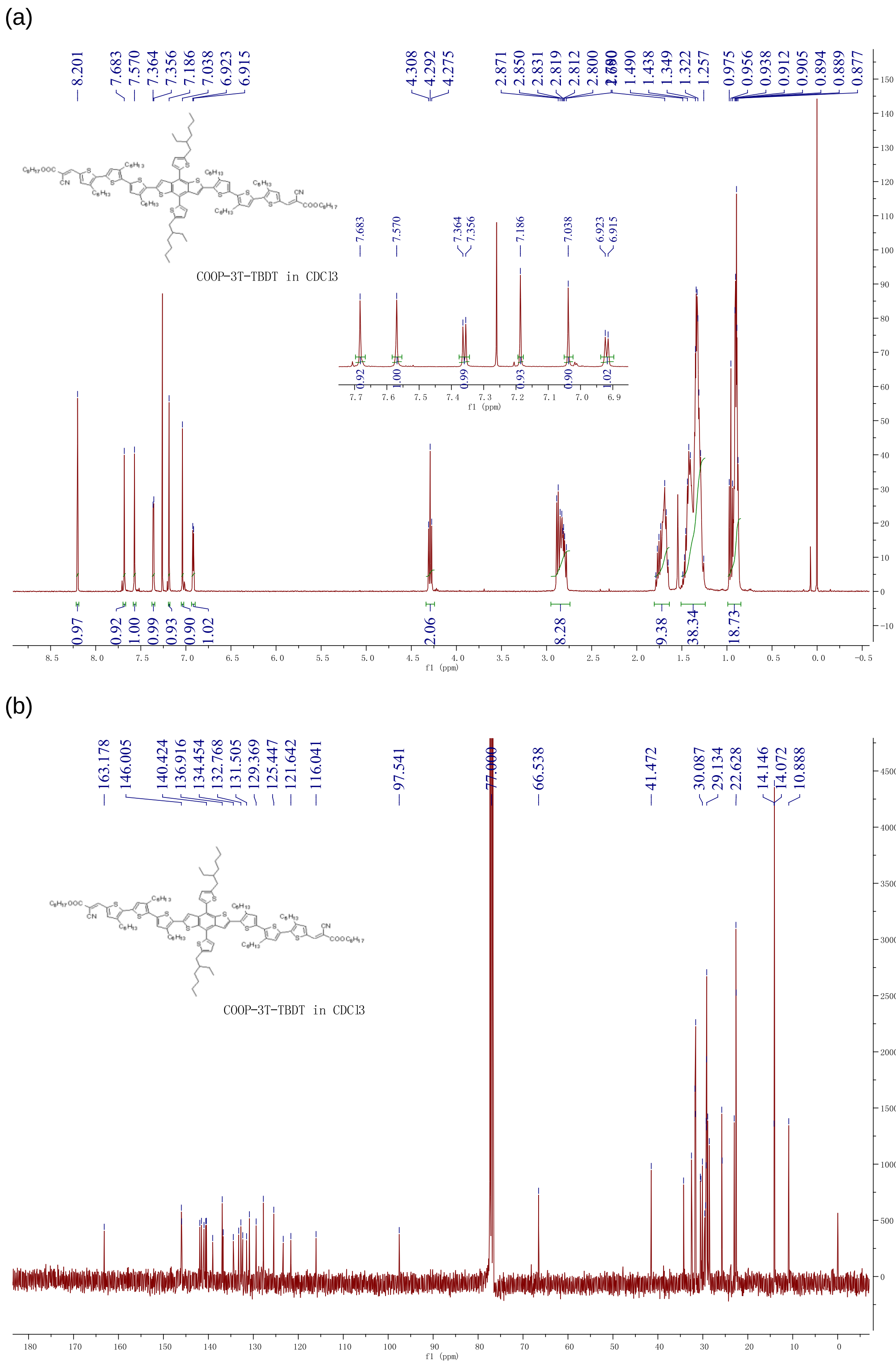

Figure S9: (a) ^{1}H and (b) ^{13}C NMR spectra of COOP-3T-TBDT ($CDCl_3$, 400MHz)

(a)

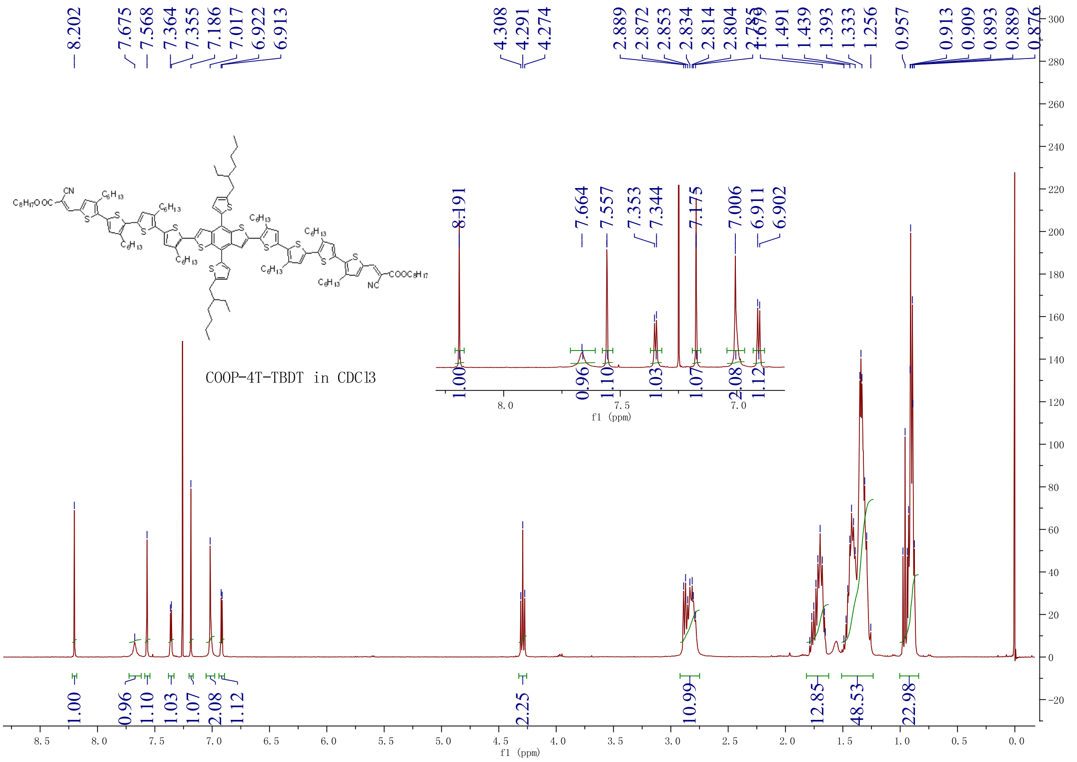

(b)

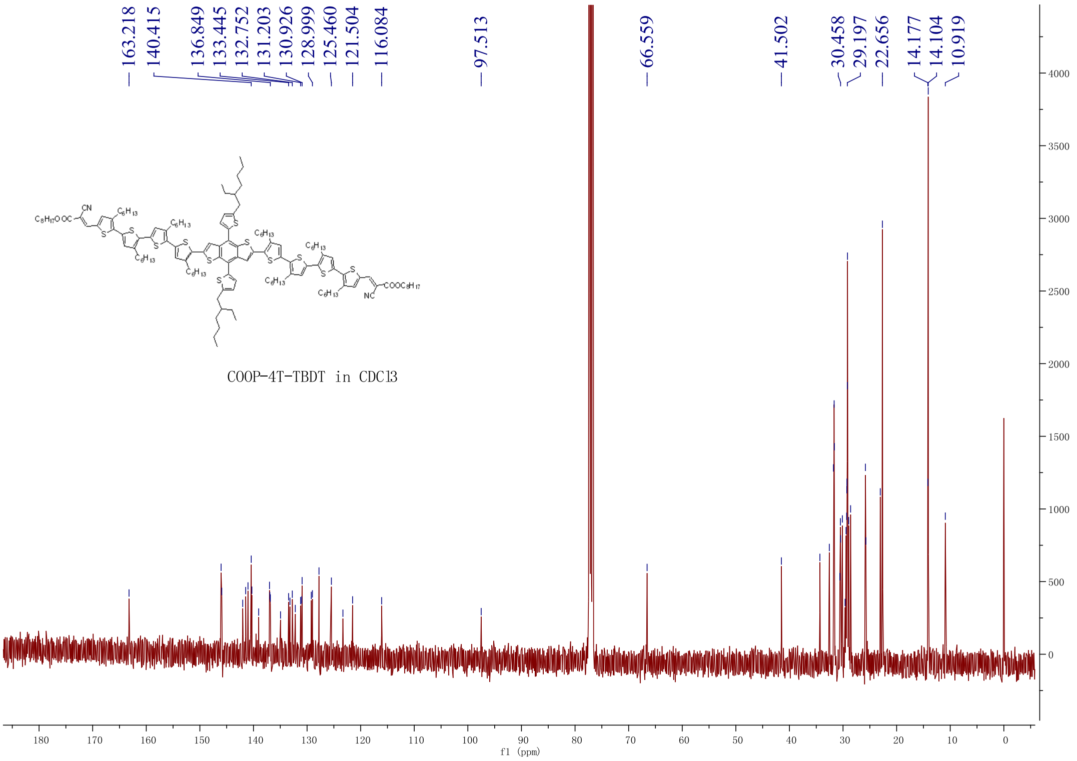

Figure S10: (a) ^{1}H and (b) ^{13}C NMR spectra of COOP-4T-TBDT ($CDCl_3$, 400MHz)

MALDI-TOF MS results

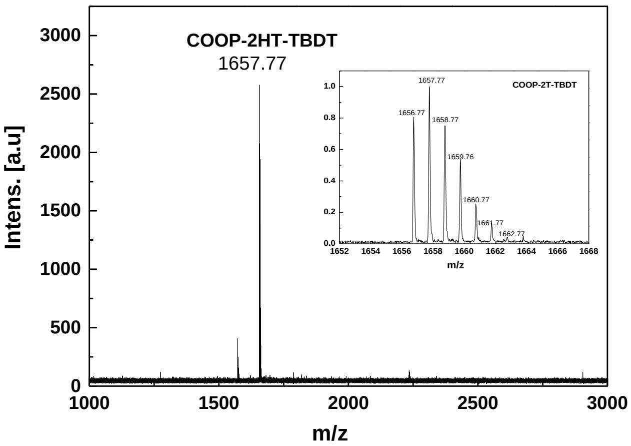

Figure S11: MS (MALDI-TOF) spectrum of **2**.

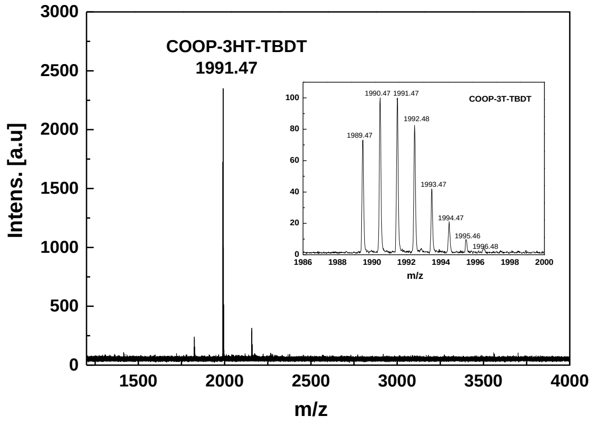

Figure S12: MS (MALDI-TOF) spectrum of **3**.

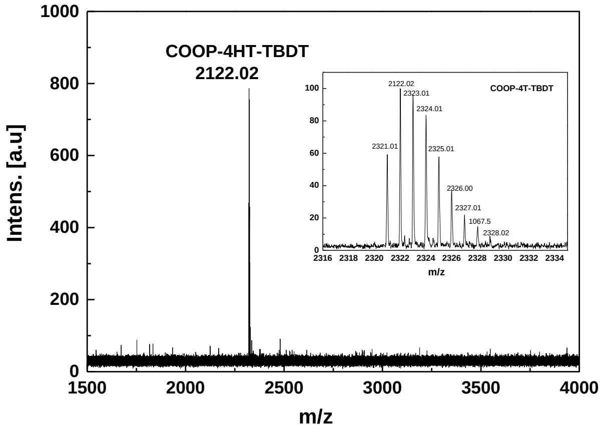

Figure S13: MS (MALDI-TOF) spectrum of **4**.
